# Investigation of genetic diversity using molecular and biochemical markers associated with powdery mildew resistance in different flax (*Linum usitatissimum* L.) genotypes

**DOI:** 10.1186/s12870-024-05113-5

**Published:** 2024-05-17

**Authors:** Marwa M. Ghonaim, Marian M. Habeb, Mahmoud T. M. Mansour, Heba I. Mohamed, Ahmed A. A. Omran

**Affiliations:** 1 Cell Study Research Department, Agriculture Research Center, Field Crops Research Institute, Giza, Egypt; 2 Plant Pathology Research Institute, Agricultural Research Center, Giza, Egypt; 3 Department of Biological and Geological Sciences, Faculty of Education, Ain Shams University, Cairo, Egypt

**Keywords:** Biochemical marker, Flax, Flavonoides, Malondialdehyde, Molecular marker, Phenolics

## Abstract

Under greenhouse conditions, the resistance of 18 different genotypes of flax to powdery mildew was evaluated. To investigate genetic diversity and identify the molecular and biochemical markers linked to powdery mildew resistance in the tested genotypes, two molecular marker systems—start codon targeted (SCoT) and inter-simple sequence repeat (ISSR)—as well as a biochemical marker (protein profiles, antioxidant enzyme activity, and secondary metabolites) were used. Based on the results, the genotypes were classified into four categories: highly susceptible, susceptible, moderately susceptible, and moderately resistant. The genotypes differed significantly in powdery mildew severity: Polk had a severity of 92.03% and Leona had a severity of 18.10%. Compared to the other genotypes, the moderately resistant genotypes had higher levels of flavonoids, antioxidant enzymes, phenolics, and straw yield; nevertheless, their hydrogen peroxide and malondialdehyde levels were lower. Protein profiles revealed 93.75% polymorphism, although the ISSR marker displayed more polymorphism (78.4%) than the SCoT marker (59.7%). Specific molecular and biochemical markers associated with powdery mildew resistance were identified. The 18 genotypes of flax were divided into two major clusters by the dendrogram based on the combined data of molecular markers. The first main cluster included Leona (genotype number 7), considered moderate resistance to powdery mildew and a separate phenetic line. The second main cluster included the other 17 genotypes, which are grouped together in a sub-cluster. This means that, besides SCoT, ISSR markers can be a useful supplementary technique for molecular flax characterization and for identifying genetic associations between flax genotypes under powdery mildew infection.

**Supplementary Information:**

The online version contains supplementary material available at 10.1186/s12870-024-05113-5.

## Introduction

Family Linaceae comprises of 22 genera of which genus *Linum* is the most wellknown. The more than 200 species present in the genus *Linum* are divided in five subsections, of which subsection *Linum* contains the cultivated species *L. usitatissimum* and the ornamentals *L. grandiflorum* and *L. perenne* [1]. Flax was already grown 6000–8000 years ago in Egypt and Sumaria, and belongs (together with barley and wheat) to the oldest of cultivated plants. Flax considered Egypt’s most ancient fiber crop after cotton, which is grown in the winter. The export and local industries of the flax crop contribute significantly to the country’s economy. One of the key crops grown in Egypt is flax, an annual diploid plant species mainly thought to be inbreeding [2]. Varietal variations in production and quality were found by numerous researchers in various flax-growing locations [3]. The fifth-largest oil crop and the world’s third-largest source of natural fiber is flax, also known as linseed or common flax. Since flax is a tiny, annual herb with self-pollinating characteristics, it can serve as a model plant for the best fiber plants [4].

One of the most significant flax diseases is powdery mildew, which is especially detrimental to the plant’s yield and oil or fiber quality. The obligate biotrophic ascomycete *Oidium lini* is the cause of this disease, which is spreading throughout Egypt. Genetic resistance among genotypes is essential to the management of the disease [5]. A major biotic stressor influencing the productivity of flax is fungus infestation. Several diseases can harm flax, but powdery mildew is one of the most common ones worldwide [5]. Powdery mildew (PM) of flax is caused by *Oidium lini* Skoric and is currently thought to be the most prevalent, noticeable, extensive, and easily identifiable foliar disease of flax in Egypt. The stems, leaves, flowers, and capsules of flax are among the aboveground organs that are infected by this fungus [4]. The airborne disease powdery mildew results in a covering of white mycelium on the surfaces of leaves and stems, which reduces photosynthetic activity and accelerates plant maturation. This caused losses in fiber and seed yield [6]. Powdery mildew appears in all Egyptian regions, and flax is produced annually. Over the last two decades, the importance of this disease has increased, probably due to the appearance and rapid distribution of new strains capable of attacking the resistant genotypes [4].

Pathogen infection can result in significant metabolic changes that are detrimental to the health of plants. Plant development and yield might be decreased by diseases [7]. Since specific gene(s) regulate the synthesis of the enzymes that govern biochemical reactions, changes in the activity and/or quantity of protein patterns would indicate changes in the gene expression pattern and related metabolic processes in the cell [8]. The production of secondary metabolites, such as phenolics, lignin, suberin, phytoalexins, alkaloids, terpenes, glycosides, degrading enzymes, and pathogenesis-related proteins, all contributed to disease resistance and genetically controlled [9]. Plants adapt to pathogen infection through the production of defense enzymes such as phenylalanine ammonia-lyase (PAL), peroxidase (POD), catalase (CAT), superoxide dismutase (SOD), and polyphenol oxidase (PPO) [10]. Reactive oxygen species, such as hydrogen peroxide (H_2_O_2_), hydroxyl radicals (OH), and superoxide anion radicals (O_2_^−^), are produced in massive amounts by pathogen-infected host plants, and these species damage the plant cell membrane [10]. The defense enzymes (SOD, CAT, and POD) play a crucial role in scavenging the oxide, which preserves the stability of the plant cell membrane and decreases the severity of plant diseases [10]. Quinones can be produced by the oxidation of monophenols and diphenols by polyphenol oxidase. It has poisonous and inhibiting effects on pathogens. The study of disease physiology is a significant indicator and a key player in improving plant disease resistance [11]. Phenylalanine ammonia-lyase enzymes are intimately linked to the host’s ability to withstand disease. It contributes to the metabolism of phenylpropanes and helps plants produce secondary biomass that is resistant to disease, including phenols, lignin, phytoalexins, and chlorogenic acids [12]. Malondialdehyde (MDA) is one of the most important consequences of membrane lipid peroxidation. Its accumulation could exacerbate membrane damage. MDA is a common marker of plant senescence and resistance. Consequently, MDA can be used to quantify the degree of membrane lipid peroxidation, which in turn provides an indirect indicator of the severity of membrane damage and the resilience of the host plants. Disease resistance is correlated with the MDA content [9].

Fungicide treatment is a typical procedure used to reduce the severity of diseases on flax. This treatment stops the powdery mildew from spreading. A progressive reduction in the application of fungicides for disease management may result from their high cost and the environmental issues they raise [13]. Excessive usage of fungicides can harm human and animal health and the environment. An alternate, environmentally friendly method of preventing the spread of powdery mildew pathotypes is breeding of resistant –disease genotypes. However, over time, genotypes of flax may lose their resistance to the diseases [14]. Thus, it will be possible to identify the most promising resistant genotypes against powdery mildew pathotypes by continuously evaluating the flax germplasms with their extremely variable genotypes. To have promising resistant genotypes for various powdery mildew pathotypes, phenotypic screening for resistance to the disease is required in large flax germplasm [4]. Selection solely based on phenotypic diversity may be deceptive because disease resistance is typically assessed using a visual score that is subject to human error [2, 3]. Thus, biochemical and molecular markers effectively substitute the powdery mildew resistance gene in the indirect selection process. It makes selection possible quickly during the growth season, greatly reducing the length of the breeding process [15].

Genetic diversity is often measured using a range of methods, such as DNA analysis (molecular markers), biochemical protein analysis (SDS-PAGE, isozyme assay), and qualitative and quantitative morphological and agronomic analysis [16]. Biochemical markers are necessary for genotype identification and the evolution of genetic variability due to their efficiency and ease of use in identifying the genetic structure of crop germplasm [17]. In addition, it is an inexpensive, straightforward, and broadly useful technique for producing an accurate genetic diversity index and showing plant protein profiles under different conditions [16].

Several crop breeding programs have included the use of a variety of molecular markers, including Simple Sequence Repeats (SSRs), Restriction Fragment Length Polymorphisms (RFLPs), Amplified Fragment Length Polymorphisms (AFLPs), and Random Amplified Polymorphic DNAs (RAPDs) [15, 18]. As a cost-effective, informative, and efficient tool, the start codon targeted (SCoT) marker is one of the most dependable approaches. Using the short-conserved area surrounding the ATG translation start codon as a guide, primers are built for this mechanism [19]. Inter simple sequence repeats (ISSRs) have several benefits, including high polymorphism, high repeatability, low DNA requirements, ease of handling, high genomic distribution, and a great potential to support breeding programs selection by identifying desirable genotypes independent of context variation [15, 20].

This study aimed at assess the response of some flax genotypes to powdery mildew infection, investigate the potential use of SCoT and ISSR approaches, use morphological and biochemical markers such as secondary metabolites and protein electrophoresis to distinguish the susceptible and resistant of flax genotypes.

## Materials and methods

### Reaction of flax genotypes to powdery mildew

**Fig. 1:**
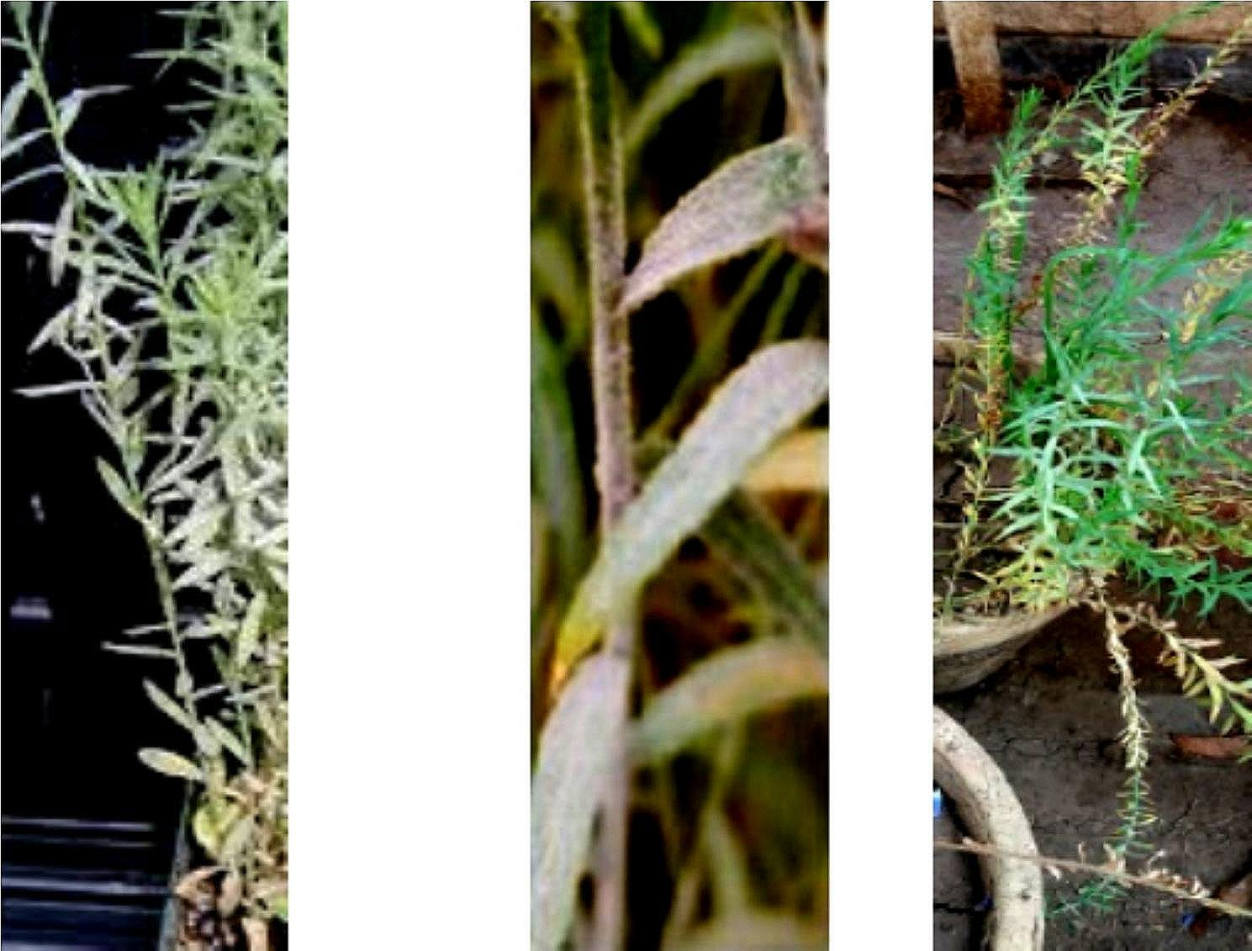
The symptoms of powdery mildew on flax

During the 2020–2021 growing season, an outdoor pot experiment was carried out at the Giza Agricultural Research Station to assess the response of 18 different genotypes of flax to powdery mildew (Table 1). From the collection of flax, germplasm kept at the Cotton and Fiber Crops Diseases Research Section, Plant Pathology Research Institute, Agricultural Research Centre, Giza, Egypt. Seeds of flax genotypes were sown in 50 cm diameter clay pots (20 seeds per pot) on 20 November 2020 under a natural soil. The pots were arranged in a randomized complete block design with five replicates to each genotype (blocks). In the last week of April 2021, powdery mildew was allowed to develop naturally (Fig. 1). The proportion of diseased leaves per plant in a randomly selected sample of ten plants per pot was used to grade the disease’s severity [21] visually. Since the surface of the infected leaf was nearly entirely covered with fungal growth at this point in the disease’s development, the disease severity was assessed based on the percentage of infected leaf area.

**Table 1:** Pedigree, name and identified resistance gene of the 18 flax genotypes

Genotypes number	Entry name	Pedigree	Identified resistance gene
1	Stewart	B12 × C.I 708	L2
2	Polk	B12 × C.I 1191	N1
3	Birio	B12 × C.I 1085	L6
4	Kenya	B7 × C.I 709	L4
5	Akmolinsk ACC	B10 × C.I 515	P1
6	Abyssinian Brown	B4 × C.I 700	P2
7	Leona	B13 × C.I 836	P3
8	Willaton Brown	B12 × C.I 803	M1
9	Victory	B12 × C.I 1170	M4
10	Bison	Bison	L9
11	B. Golden Sel	B6 × C.I 1183	L10
12	Barnes	B6 × C.I 1190	L7
13	Bisbee	B12 × C.I 13,360	L8
14	Pale Blue Crimped	B8 × C.I 647	L3
15	Burk	B7 × C.I 1180	L1
16	Ward	B14 × C.I 1181	M2
17	B6 × Kugler C	B6 × Kugler C	Kugen
18	Linora	Linora	L11

### Determination of antioxidant enzymatic activities

One gram of flax shoots was pulverized in 250 µL of 50 mM (0.1 M, pH 6.5) sodium phosphate buffer, and the mixture was centrifuged for 30 min at 4 °C and 8500 rpm. A supernatant was utilized to identify antioxidant enzymes.

A mixture of 25 mM phosphate buffer (pH 6.8), 0.1 mM pyrogallol, and the supernatant (0.1 mL) was used to measure the amount of polyphenol oxidase enzyme (PPO; EC 1.1418.1). Using a spectrophotometer, the mixture was measured at 525 nm in accordance with the Mayer procedure [22]. Peroxidase enzyme (POD; EC 1.11.1.7) was measured using the approach of Pütter [23]. A mixture of 40 µL of 0.1% H_2_O_2_ and 0.1 mL of 20 mM guaiacol was added to the 100 µL of supernatant. The absorbance of the mixture was read at 470 nm using a spectrophotometer.

A method for measuring the catalase enzyme (CAT; EC 1.11.1.6) was developed by Ramalingam and In-Jung [24]. 6 mL of the reaction mixture, 1.0 mL of 75 mM H_2_O_2_, and 3.0 mL of 0.2 mM phosphate buffer (pH 7) were added. Additionally, 100 µL of diluted enzyme extract was added. Using a spectrophotometer to measure the mixture’s absorbance at 240 nm, the amount of H_2_O_2_ decomposed allowed for the calculation of the enzyme activity.

Superoxide dismutase enzyme (SOD; EC 1.15.1.1) was measured using the following solutions: 13.33 mM methionine, 75 µM NBT, 0.1 mM EDTA, 50 mM phosphate buffer (pH 7.8), 50 mM sodium carbonate, 0.1 mL enzyme extract, and 2 mM riboflavin (0.1 mL). The tubes were then exposed to two 15 W fluorescent lamps for 15 min to begin the reaction. Upon measuring the absorbance at 560 nm with a spectrophotometer, the sample was allocated one unit of enzyme activity, leading to a 50% reduction in absorbance compared to tubes lacking enzyme [25].

The activity of phenylalanine ammonia lyase (PAL; EC 4.3.1.24) was determined by utilizing a standard trans-cinnamic acid curve to measure the trans-cinnamic acid production at 290 nm using a spectrophotometer according to McCallum and Walker [26] method. The enzyme reaction mixture included an aliquot of the enzyme in a volume of 1 mL, 40 mM _L_-phenylalanine, and 100 mM Tris-HCl incubated at 37 °C for 30 min, and the reaction was stopped by adding 50 µL of 4 M HCl.

### Determination of lipid peroxidation

Thiobarbituric acid (TBA) test measured MDA content [27]. A half gram of flax shoots was extracted using 5 mL of 5% trichloroacetic acid and centrifuged at 3,000 ×g for 10 min. Two mL of the supernatant was mixed with 2 mL of 0.67% (w/v) TBA and heated in a water bath for 30 min. The optical density of the mixture was read using a spectrophotometer at 450, 532, and 600 nm. The MDA concentration was calculated using an extinction value of 155 mM cm^− 1^.

### Determination of hydrogen peroxide (H_2_O_2_)

Using the Xylenol orange method, the quantity of H_2_O_2_ in 0.1% TCA extracts of flax shoot samples was determined [28]. Flax shoots (0.5 g) were homogenized in 0.1% TCA. The homogenate was filtered following its preparation. 0.5 mL of leaf extract was combined with 2 mL of reagent (1 M KI w/v) and 0.5 mL of 100 mM K-phosphate buffer (pH 6.8). Using a spectrophotometer, the absorbance of the reaction was measured at 390 nm after an hour.

### Determination of secondary metabolites content

Total phenolic and flavonoid contents were determined by extracting dry flax shoots from different genotypes three times at 90 °C in 80% cold methanol. The absorbance of the mixture was measured at 760 nm following filtration by the Dihazi et al. [29] method, following the addition of phenol and ferric chloride to the filtrate. The total phenolic contents were calculated using the gallic acid standard curve and represented as mg gallic acid g^− 1^. To calculate the flavonoid amount, the filtrate was also combined with NaNO_2_ and 10% w/v of AlCl_3_, and the optical density of the mixture was measured at 510 nm using a spectrophotometer according to the Ghasemzadeh et al. [30] method.

### SDS-PAGE analysis

Protein electrophoresis pattern of harvested seeds on 12% polyacrylamide gels was determined using the technique outlined by Laemmli [31] and modified by Studier [32]. 0.25% (w/v) Coomassie Brilliant Blue R-250 was used to fix and stain the gel after electrophoresis. The gel was photographed, scanned, and analyzed using the Gel Doc 2000 Bio-Rad apparatus.

### Genomic DNA isolation

Genomic DNA was isolated from flax leaves of different genotypes according toAshry et al. [8]. The following ingredients were used to produce cetyltrimethylammonium bromide (CTAB) solution: 2% CTAB, 1.5 M NaCl, 10 mM Na_3_EDTA, 0.1 M HEPES-acid, 100% isopropanol (isopropyl alcohol, 2-propanol), 70% ethanol, and 1xTE (10 mM of Tris-HC1, pH 8.0; 1 mM of EDTA).

### **PCR amplification of ISSR and SCoT markers**

In this study, 23 primers (12 SCoT and 11 ISSR) were used (Table 2). For both markers, PCR was performed in 20 µL using 10X PCR buffer, 25 mM MgCl_2_, 10 mM dNTPs, 2 µM primers, 5 U Taq DNA polymerase, and 100 ng of template DNA. Every PCR reaction was run through a Perkin Elmer and Eppendorf thermal cycler. 95 °C for 5 min; 35 cycles (95 °C for 30 s, melting temperature (Tm) 45–60ºC for 45 s, 72 °C for 1:30 min) and 72 °C for 5 min were programmed for the PCR. The amplification products were separated by electrophoresis in a 1.2% agarose gel in 1X THE buffer at 80 V with ethidium bromide (0.5 µg/mL). PCR products underwent UV light observation and were captured on camera with a Gel Documentation System (BIO-RAD 2000).

**Table 2:** List of SCOT and ISSR primers and their sequence, G-C content and melting temperature (Tm)

Primer cod Sequence	Primer cod Sequence	%GC
**SCOT primer**		
SCOT 1	CAACA ATGGCTACCACCA	50
SCOT 6	CAACA ATGGCTACCACGC	56
SCOT 9	CAACA ATGGCTACCAGCA	50
SCOT 11	AAGCA ATGGCTACCACCA	50
SCOT 12	ACGAC ATGGCGACCAACG	61
SCOT13	ACGAC ATGGCGACCATCG	61
SCOT 21	ACGAC ATGGCGACCCACA	61
SCOT 22	AACC ATGGCTACCACCAC	56
SCOT 25	ACC ATGGCTACCACCGGG	67
SCOT 34	ACC ATGGCTACCACCGCA	61
SCOT 35	C ATGGCTACCACCGGCCC	72
SCOT 36	GCAACA ATGGCTACCACC	56
**ISSR Primers**		**Tm°C**
UBC 814	CTC TCT CTC TCT CTC AT	50
UBC 686	GAA GAA GAA GAA GAA GAA	47
UBC 826	ACA CAC ACA CAC ACA CC	52
UBC 827	ACA CAC ACA CAC ACA CG	52
UBC 811	GAG AGA GAG AGA GAG C	51
ISSR 9 (UBC 901)	CAC ACA CAC ACA CAC ARY	54
ISSR 10(ISSR 810)	GAG AGA GAG AGA GAG AT	50
ISSR 835	AGA GAG AGA GAG AGA GYC	55
ISSR 825	ACA CAC ACA CAC ACT	43
ISSR 807	AGA GAG AGA GAG AGA GT	50
ISSR 857	ACA CAC ACA CAC ACA CYG	55

### Polymorphism information content (PIC)

According to Botstein et al. [33], the measure or value of the polymorphism information content (PIC) is based on a marker’s capacity to establish polymorphism in the population based on the number of alleles found and the frequency of their distribution. The value of PIC can be calculated by this Eq. (1):

1 $$\text{PIC}={1}-{\sum}^{n}{f}^2{i},\,{i}={1}$$

Where${f}^2{i}$ is the frequency of the *i* allele (presence of band) and *n* is the total number of loci in the flax-tested genotypes. PIC values were between 0 (monomorphic) and 1 (very highly discriminative, with numerous alleles occurring at low and equal frequency).

### Data analysis

The band profiles of SCoT and ISSR primers were distinguished as present (1) or absent (0) rating for distinct. The level of genetic relatedness between genotypes in the present study was assessed by analyzing the banding patterns of 12 SCoT and 11 ISSR primers. The Unweighted Pair Group Method with Arithmetic Averages (UPGMA) and SAHN (Sequential, Agglomerative, Hierarchical, and Nested Clustering) algorithms from the NTSYS-PC (Numerical Taxonomy and Multivariate Analysis System), version 2.1 (Applied Biostatistics) program were used to calculate the similarity coefficients [34]. A principal component analysis (PCA) based on the SCoT and ISSR data matrix was also produced using PAST software 4.02. Heat maps were created using ClustVis, an online application for visualizing the clustering of multivariate data [35].

### Statistical analysis

The pots were arranged in a randomized complete block design of five replicates to each genotype. Means of three replicates were taken. R software version 2.14.1 was utilized to conduct the statistical analysis. To identify significant differences among the examined morphological and biochemical parameters (antioxidant enzymes, lipid peroxidation, hydrogen peroxide and secondary metabolites), Duncan’s Multiple Range Test (DMRT) was employed. The significance level was set at *p* ≤ 0.05. ANOVA (Analysis of Variance) was performed using [36], by the procedure outlined by Gomez and Gomez [37].

## Results

### Effect of powdery mildew on disease severity

Environmental conditions in the 2020–2021 growing seasons, such as warm temperatures and high relative humidity, favored powdery mildew development. The highest disease severity was detected in genotypes 1, 2, 4, 8, 10, 11, 12, and 13 by about 89.51, 92.03, 79.84, 78.22, 81.91, 81.40, 84.82, and 85.64%, respectively. These genotypes are considered to be highly susceptible to powdery mildew. In addition, the tested genotypes can be classified into four groups according to the severity of powdery mildew disease: highly susceptible (Stewart, Polk, Kenya, Willaton Brown, Bison, B. Golden Sel, Barnes, and Bisbee genotypes), susceptible (Birio, Akmolinsk ACC, Bison, Pale Blue Crimped, Ward, and Linora genotypes), moderately susceptible (Abyssinian Brown, Burk, and B6 x Kugler C genotypes), and moderately resistant (Leona genotype). The genotypes showed considerable variation in powdery mildew severity, ranging from 18.10% on the Leona genotype to 92.03% on the Polk genotype (Table 3).

**Table 3:** Powdery mildew severity on the 18 flax genotypes evaluated in outdoor pot experiment

Genotypes number	Genotypes name	Severity (%)	Seed weight (mg)	Straw weight (mg)	Reaction class ^b^
1	Stewart	89.51 ± 2.1^a^	111.75 ± 3.4^bc^	468.09 ± 6.5^b^	HS
2	Polk	92.03 ± 2.5^a^	111.99 ± 4.0^bc^	349.54 ± 5.1^c^	HS
3	Birio	54.44 ± 1.6^c^	119.50 ± 3.5^b^	445.78 ± 4.5^b^	S
4	Kenya	79.84 ± 2.9^ab^	135.83 ± 3.6^b^	355.28 ± 3.3^c^	HS
5	Akmolinsk ACC	68.17 ± 2.0^ab^	129.84 ± 3.5^b^	369.70 ± 3.9^c^	S
6	Abyssinian Brown	32.56 ± 1.3^e^	146.34 ± 2.9^ab^	561.75 ± 7.2^a^	MS
7	Leona	18.10 ± 1.5^e^	172.55 ± 4.2^a^	580.74 ± 5.6^a^	MR
8	Willaton Brown	78.22 ± 2.6^ab^	86.65 ± 3.3^cd^	385.21 ± 4.2^c^	HS
9	Victory	53.62 ± 2.5^d^	98.14 ± 2.6^c^	450.19 ± 4.5^b^	S
10	Bison	81.91 ± 2.9^ab^	94.01 ± 2.1^c^	413.89 ± 4.6^b^	HS
11	B. Golden Sel	81.40 ± 2.0^ab^	101.39 ± 2.6^c^	319.06 ± 3.9^c^	HS
12	Barnes	84.82 ± 3.2^ab^	69.29 ± 1.5^d^	351.10 ± 3.^8^	HS
13	Bisbee	85.64 ± 3.4^ab^	92.71 ± 2.9^c^	439.41 ± 4.1^b^	HS
14	Pale Blue Crimped	68.59 ± 2.7^bc^	109.97 ± 2.9^c^	454.19 ± 4.3^b^	S
15	Burk	38.94 ± 1.6^d^	153.20 ± 4.1^ab^	560.50 ± 5.7^a^	MS
16	Ward	73.26 ± 3.2^b^	111.72 ± 3.1^bc^	456.89 ± 4.5^b^	S
17	B6 × Kugler C	47.46 ± 1.6^d^	136.96 ± 2.8^b^	419.07 ± 5.0^b^	MS
18	Linora	74.08 ± 3.2^b^	37.11 ± 1.3e	457.16 ± 5.0^b^	S

Mean values of three replicates and standard deviation (± SD) in each column followed by a different lower-case letter are significantly different according to Duncan’s multiple range tests at *p* ≤ 0.05

Reaction classes of the tested genotypes were determined based on disease severity according to the following scale: Highly resistant (HR) = 0, Resistant (R) = 1–10, Moderately resistant (MR) = 11–25, Moderately susceptible (MS) = 26–50, Susceptible (S) = 51–75, Highly susceptible (HS) = 76–100 [38].

### Effect of powdery mildew on straw yield and seed yield

The straw yield and seed yield of the moderately resistant genotype (Leona) were higher than those of the other genotypes (Table 3). However, the straw and seed yields of the highly susceptible genotypes were lower than the other genotypes.

### Effect of powdery mildew on antioxidant enzymes

The data in Table 4 shows the differences in the antioxidant enzyme activity of flax genotypes. The antioxidant enzymes (PPO, POD, CAT, SOD, and PAL) in moderately resistant genotype number 7 (Leona) were significantly higher than the other genotypes. Genotypes number 6 (Abyssinian Brown), 15 (Burk), and 17 (B6 x Kugler C), which are considered moderately susceptible to powdery mildew, have higher antioxidant enzymes than the other genotypes.

**Table 4:** Effect of powdery mildew on antioxidant enzymes of flax genotypes shoots

Genotypes number	Genotypes name	Polyphenol oxidase enzyme (PPO) (nmol min^− 1^ g^− 1^ FW)	Peroxidase (POD) (nmol min^− 1^ g^− 1^ FW)	Catalase (CAT) (nmol min^− 1^ g^− 1^ FW)	Superoxide dismutase (SOD) (nmol min^− 1^ g^− 1^ FW)	Phenylalanine ammonia lyase (PAL) (mmol Trans Cinamic acid min^− 1^ g^− 1^ FW)
1	Stewart	15.5 ± 0.1^g^	9.6 ± 0.1^g^	18.9 ± 0.2^e^	10.1 ± 0.1^h^	3.3 ± 0.05^h^
2	Polk	14.8 ± 0.1^h^	12.3 ± 0.1^e^	19.6 ± 0.2^e^	12.3 ± 0.1^g^	4.5 ± 0.02^g^
3	Birio	19.5 ± 0.2^cd^	11.5 ± 0.1^f^	25.6 ± 0.2^c^	17.4 ± 0.1^cd^	6.2 ± 0.02^ef^
4	Kenya	15.6 ± 0.1^g^	14.6 ± 0.2^c^	17.5 ± 0.3^f^	11.8 ± 0.1 g	6.9 ± 0.03^e^
5	Akmolinsk ACC	18.9 ± 0.2^d^	14.3 ± 0.1^c^	20.2 ± 0.3^e^	16.5 ± 0.2^d^	5.5 ± 0.02^f^
6	Abyssinian Brown	20.2 ± 0.3^c^	16.1 ± 0.1^b^	33.4 ± 0.4^b^	20.1 ± 0.2^b^	10.2 ± 0.05^b^
7	Leona	23.3 ± 0.3^a^	18.9 ± 0.2^a^	38.9 ± 0.4^a^	22.5 ± 0.2^a^	13.3 ± 0.05^a^
8	Willaton Brown	13.2 ± 0.1^i^	10.1 ± 0.1^g^	16.3 ± 0.2^f^	14.9 ± 0.1^e^	7.0 ± 0.02^e^
9	Victory	22.1 ± 0.2^b^	15.8 ± 0.1^b^	21.2 ± 0.2^d^	18.9 ± 0.2^b^	8.9 ± 0.02^cd^
10	Bison	15.6 ± 0.2^g^	12.2 ± 0.1^e^	17.9 ± 0.1^f^	15.6 ± 0.1^d^	8.1 ± 0.02^d^
11	B. Golden Sel	15.5 ± 0.1^g^	14.6 ± 0.2^c^	17.1 ± 0.1^f^	13.5 ± 0.1^f^	5.5 ± 0.02^f^
12	Barnes	13.3 ± 0.1^i^	13.3 ± 0.1^d^	18.9 ± 0.1^ef^	15.3 ± 0.1^de^	7.8 ± 0.02^d^
13	Bisbee	16.4 ± 0.1^f^	12.5 ± 0.1^e^	16.9 ± 0.1^f^	12.6 ± 0.1^g^	4.9 ± 0.01^f^
14	Pale Blue Crimped	14.3 ± 0.1^h^	13.3 ± 0.1^d^	23.6 ± 0.3^d^	17.9 ± 0.1^c^	10.7 ± 0.03^b^
15	Burk	17.5 ± 0.2^e^	15.6 ± 0.2^b^	25.8 ± 0.2^c^	18.6 ± 0.2^c^	9.3 ± 0.03^c^
16	Ward	15.0 ± 0.1g^h^	14.2 ± 0.1^c^	24.2 ± 0.2^d^	18.0 ± 0.2^c^	7.6 ± 0.02^d^
17	B6 x Kugler C	16.5 ± 0.1^f^	15.8 ± 0.2^b^	26.4 ± 0.2^c^	19.0 ± 0.2^b^	9.1 ± 0.02^c^
18	Linora	17.2 ± 0.1^ef^	11.5 ± 0.1^f^	22.6 ± 0.3^d^	14.8 ± 0.1	6.4 ± 0.01^e^

Mean values of three replicates and standard deviation (± SD) in each column followed by a different lower-case letter are significantly different according to Duncan’s multiple range tests at *p* ≤ 0.05

### Effect of powdery mildew on oxidative stress and secondary metabolites

The MDA and H_2_O_2_ content in the shoots of the moderately resistant genotypes (Leona) were lower than that in the shoots of the other genotypes (Table 5). In addition, the MDA and H_2_O_2_ content in the highly susceptible genotypes were higher than that in the other genotypes.

**Table 5:** Effect of powdery mildew on oxidative stress and secondary metabolites of flax genotypes shoots

Genotypes number	Genotypes name	Lipid peroxidation (nmol MDA g^− 1^ FW)	Hydrogen peroxide (µmol g^− 1^ FW)	Phenol (µg gallic acid /g DW)	Flavonoids (µg querectin/g DW)
1	Stewart	15.75 ± 0.1^g^	6.10 ± 0.03^b^	25.25 ± 0.2^e^	44.12 ± 0.2^g^
2	Polk	16.71 ± 0.2^f^	3.55 ± 0.01^e^	35.33 ± 0.2^g^	50.32 ± 0.3^f^
3	Birio	18.40 ± 0.3^ef^	5.05 ± 0.02^c^	65.22 ± 0.4^c^	61.05 ± 0.4^d^
4	Kenya	22.44 ± 0.6^c^	5.30 ± 0.02^c^	52.69 ± 0.3^e^	60.51 ± 0.5^d^
5	Akmolinsk ACC	17.90 ± 0.3^f^	4.10 ± 0.01^d^	71.30 ± 0.5^b^	69.23 ± 0.3^c^
6	Abyssinian Brown	15.00 ± 0.4^g^	4.22 ± 0.01^d^	83.44 ± 0.5^a^	72.33 ± 0.7^b^
7	Leona	9.50 ± 0.1i	2.71 ± 0.01^f^	87.20 ± 0.4^a^	77.20 ± 0.7^a^
8	Willaton Brown	12.30 ± 0.1^h^	5.30 ± 0.01^c^	42.20 ± 0.2^f^	52.14 ± 0.4^ef^
9	Victory	20.25 ± 0.3^d^	4.15 ± 0.02^d^	58.10 ± 0.3^d^	69.20 ± 0.^4c^
10	Bison	28.00 ± 0.6^a^	3.20 ± 0.02^e^	35.35 ± 0.3^g^	43.22 ± 0.3^g^
11	B. Golden Sel	19.55 ± 0.4^e^	3.00 ± 0.01^e^	50.25 ± 0.3^e^	55.65 ± 0.4^e^
12	Barnes	19.77 ± 0.2^e^	2.32 ± 0.01^e^	50.37 ± 0.2^e^	62.10 ± 0.4^d^
13	Bisbee	21.00 ± 0.4^cd^	5.50 ± 0.02^c^	34.20 ± 0.2^g^	50.23 ± 0.3^f^
14	Pale Blue Crimped	15.10 ± 0.3^g^	4.09 ± 0.01^d^	59.55 ± 0.3^d^	69.52 ± 0.3^c^
15	Burk	9.80 ± 0.1^i^	3.60 ± 0.01^e^	71.30 ± 0.4^b^	75.15 ± 0.7^a^
16	Ward	20.55 ± 0.2^de^	6.60 ± 0.02^b^	69.20 ± 0.4^b^	65.34 ± 0.4^d^
17	B6 x Kugler C	12.80 ± 0.1^h^	3.60 ± 0.01^e^	83.10 ± 0.5^a^	70.54 ± 0.6^bc^
18	Linora	24.40 ± 0.4^b^	7.70 ± 0.02^a^	67.76 ± 0.4^b^	62.44 ± 0.4^d^

Mean values of three replicates and standard deviation (± SD) in each column followed by a different lower-case letter are significantly different according to Duncan’s multiple range tests at *p* ≤ 0.05

In addition, phenolic and flavonoid contents in shoots of the moderately resistant genotypes (Leona) was higher than that in the shoots of the other moderately susceptible, susceptible, and highly susceptible genotypes. However, the phenolic and flavonoid content in the shoots of the highly susceptible genotypes was lower than that in the other genotypes (Table 5).

### SDS-PAGE of seed storage protein patterns

**Fig. 2:**
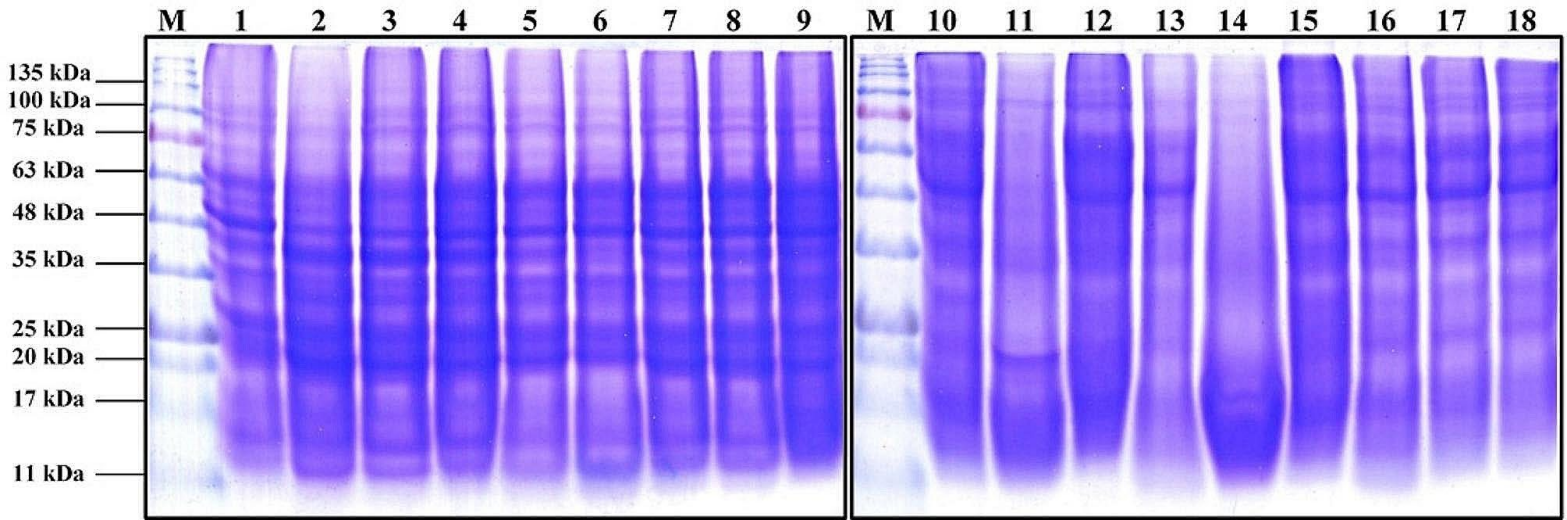
SDS banding pattern of seed protein for the 18 flax genotypes tested under biotic stress. M (protein marker), Lanes 1–18 flax genotypes. Stewart (1), Polk (2), Birio (3), Kenya (4), Akmolinsk ACC (5), Abyssinian Brown (6), Leona (7), Willaton Brown (8), Victory (9), Bison (10), B. Golden Sel (11), Barnes (12), Bisbee (13), Pale Blue Crimped (14), Burk (15), Ward (16), B6 × Kugler C (17), Linora (18)

The electrophoretic banding pattern of the harvested seeds of the 18 genotypes of flax is displayed in Fig. 2, and Table 6 gives details about SDS-PAGE scanning, band scoring, band molecular weights in kDa, and band presence or absence (1) or (0). There are 16 bands with a 93.75% polymorphism rate among the 18 flax genotypes. The molecular weights of the polypeptides ranged from 98.63 to 11.83 kDa. The Bisbee and Pale Blue Crimped genotypes (lanes 13 and 14) had the lowest number of bands, about 4 bands, while the Abyssinian Brown genotypes in lane 6 had the highest number of bands, about 15 bands. Furthermore, the final profile comprises 15 polymorphic bands and 1 monomorphic band. There were no discernible bands found.

**Table 6:** The molecular mass (Mr.) in kilo-Daltons (kDa) of the produced SDS-PAGE of seed protein bands and their presence (1) or absence (0), number and type of the bands as well as the percentage of the polymorphism detected in the 18 flax genotypes under biotic stress

No.	Rf	Mr (kDa.)	Genotypes																				
			1	2	3	4	5	6	7		8	9	10		11	12	13		14	15	16	17	18
1	0.113	98.63	1	1	1	1	1	1	1		1	1	1		0	1	0		0	1	1	1	1
2	0.155	85.43	0	0	0	0	0	0	0		0	0	1		1	0	0		1	1	0	0	0
3	0.173	81.53	1	1	1	1	1	1	1		1	1	0		0	1	1		0	0	1	1	1
4	0.224	69.51	1	1	1	1	1	1	1		1	1	0		0	0	0		0	0	0	0	0
5	0.231	65.85	0	0	0	0	0	1	0		0	0	1		0	0	0		0	0	0	0	0
6	0.270	60.12	1	1	1	1	1	1	1		1	1	0		0	1	1		0	1	1	1	1
7	0.342	50.96	1	1	1	1	1	1	1		1	1	1		1	0	0		0	0	0	0	1
8	0.366	45.86	1	1	1	1	1	1	1		1	1	1		1	1	1		0	1	1	1	0
9	0.435	40.12	1	1	1	1	1	1	1		1	1	0		0	0	0		0	0	1	1	0
10	0.473	34.04	1	1	1	1	1	1	1		1	1	1		1	1	1		1	1	1	1	1
11	0.543	30.54	0	1	1	1	1	1	1		1	1	0		0	0	0		0	0	0	1	1
12	0.606	25.81	1	1	1	1	1	1	1		1	1	1		0	0	0		0	1	1	0	1
13	0.678	20.72	1	1	1	1	1	1	1		1	1	1		1	1	0		0	1	1	1	0
14	0.791	16.82	1	1	1	1	1	1	1		0	1	1		1	1	0		1	1	1	1	1
15	0.873	14.26	1	1	1	1	1	1	1		1	1	0		1	0	0		1	0	0	0	0
16	0.946	11.83	0	1	1	1	1	1	1		1	0	0		0	0	0		0	0	0	0	0
Total number of bands			12	14	14	14	14	15	14		13	13	9		7	7	4		4	8	9	9	8
Total number of bands			Monomorphic bands							Polymorphic bands								Polymorphism (%)					
										Shared bands				Unique bands									
16			**1**							**15**				**0**				**93.75**					

M (marker), Lanes 1–18 flax genotypes. Stewart (1), Polk (2), Birio (3), Kenya (4), Akmolinsk ACC (5), Abyssinian Brown (6), Leona (7), Willaton Brown (8), Victory (9), Bison (10), B. Golden Sel (11), Barnes (12), Bisbee (13), Pale Blue Crimped (14), Burk (15), Ward (16), B6 x Kugler C (17), Linora (18)

### Molecular marker

#### SCoT analysis

**Fig. 3:**
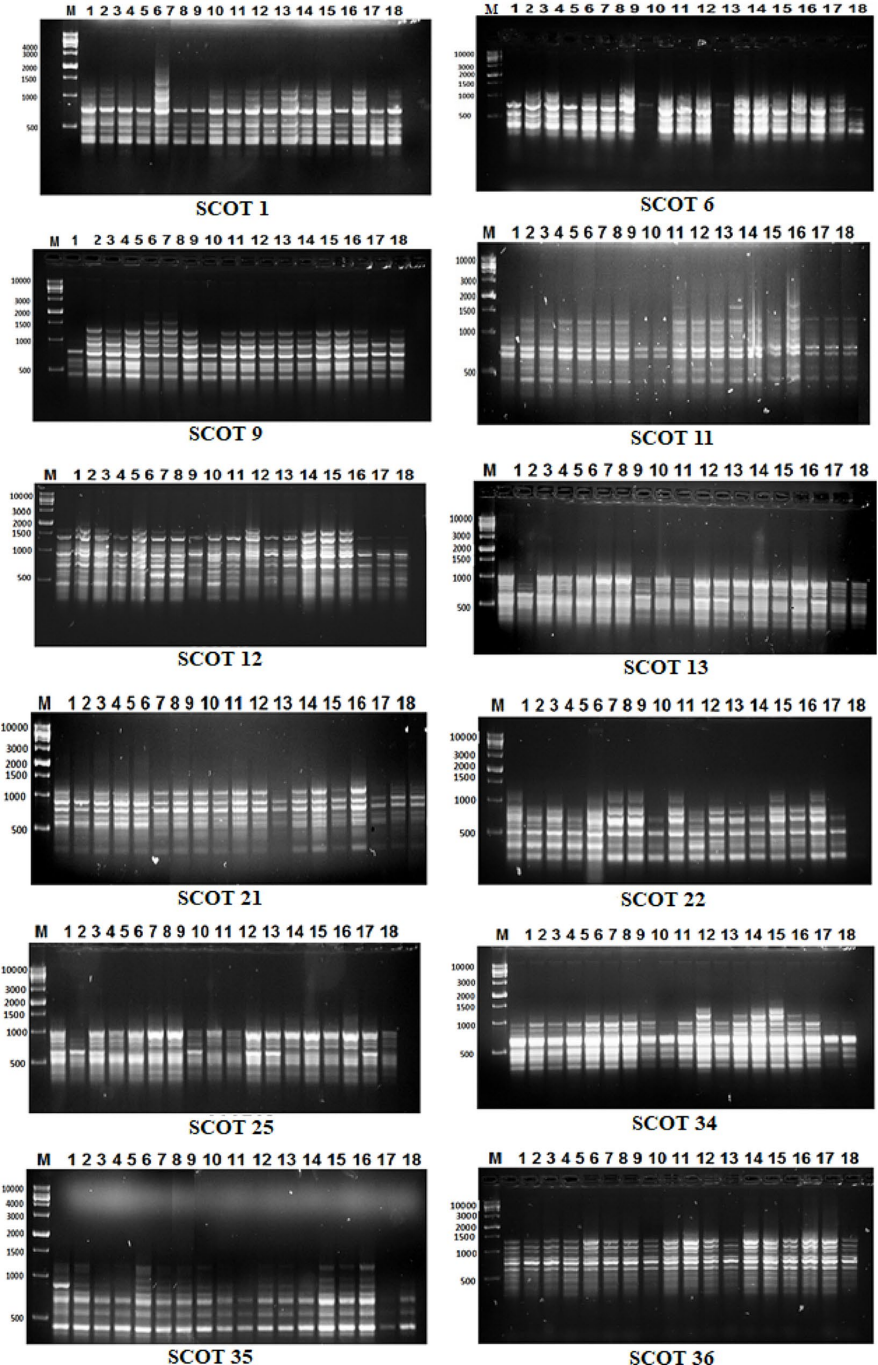
SCoT fingerprints of the ten barely genotypes tested using 12 primers. M (marker), Lanes 1–18 flax genotypes. Stewart (1), Polk (2), Birio (3), Kenya (4), Akmolinsk ACC (5), Abyssinian Brown (6), Leona (7), Willaton Brown (8), Victory (9), Bison (10), B. Golden Sel (11), Barnes (12), Bisbee (13), Pale Blue Crimped (14), Burk (15), Ward (16), B6 x Kugler C (17), Linora (18)

As shown in Fig. 3; Tables 7 and 12 SCoT primers produced a total of 119 bands; 48 bands were monomorphic, and 71 bands were polymorphic, with 59.7% (polymorphism) including 18 unique bands (6 positive specific markers and 12 negative specific markers). The molecular size ranged from 100 to 1500 bp, and the number of bands was between 5 and 14. In addition, SCoT-12 produced 14 bands, followed by SCoT-36, which produced 12 bands; SCoT-1 and SCoT-1 produced 11 bands; and SCoT-9, SCoT-11, and SCoT-35 produced 10 bands.

**Table 7:** Bands variation and polymorphism percentage for flax genotypes using SCoT primers

Primer	No. of bands	Polymorphic bands	Monomorphic bands	Polymorphism %	PIC	Unique bands	Ve + U.B	Ve- U.B	Genotypes	Band size (bp)
**SCOT primers**										
SCoT-1	11	5	6	45.5%	0.941	1	1	0	5	1300
SCoT-6	8	6	2	75.0%	0.926	2	2	0	7, 5	1100, 600
SCoT-9	10	6	4	60.0%	0.919	1	0	1	1	900
SCoT-11	10	7	3	70.0%	0.939	0	0	0	-	-
SCoT-12	14	11	3	78.6%	0.958	2	0	2	8, 10	800, 300
SCoT-13	9	4	5	44.4%	0.910	2	0	2	2	900, 400
SCoT-21	10	6	4	60.0%	0.931	1	0	1	12	100
SCoT-22	5	2	3	40.0%	0.850	0	0	0	-	-
SCoT-25	9	8	1	88.9%	0.923	5	1	4	2, 14, 5, 16, 2	1000, 800, 700, 600, 480
SCoT-34	11	6	5	54.5%	0.939	2	1	1	15, 18	1500, 400
SCoT-35	10	7	3	70.0%	0.947	1	1	0	1	950
SCoT-36	12	3	9	25.0%	0.929	1	0	1	8	400
**Total**	**119**	**71**	**48**	**-**	**-**	**18**	**6**	**12**	**-**	**-**
**Average**	**-**	**-**	**-**	**59.7%**	**0.926**	**-**	**-**	**-**	**-**	**-**

Flax genotypes: Stewart (1), Polk (2), Birio (3), Kenya (4), Akmolinsk ACC (5), Abyssinian Brown (6), Leona (7), Willaton Brown (8), Victory (9), Bison (10), B. Golden Sel (11), Barnes (12), Bisbee (13), Pale Blue Crimped (14), Burk (15), Ward (16), B6 x Kugler C (17), Linora (18)

Additionally, the primer SCoT-12, which produced 14 bands, had the highest number of polymorphic bands (11 bands). The SCoT-25 primer generated the highest number of unique bands (5). In addition, primer SCoT-22 recorded the lowest number of polymorphic fragments (5) and also produced the lowest number of unique bands (0). In addition, the highest polymorphism percentage was observed in the primers SCoT-25 (88.9%), followed by SCoT-12 (78.6%), and followed by the primer SCoT-6 (75.0%); however, the lowest polymorphism percentage was observed in the primer SCoT-36 (25.0%).

The molecular genetic distinctions between the eighteen flax genotypes were clarified by using the data shown in Table 7, which also identified the unique markers for each genotype to serve as a basis for classification.In addition,, these bands can be considered molecular genetic markers for biotic stress (powdery mildew) tolerance in these 18 flax genotypes. The primer SCoT-6 exhibited one positive specific marker of powdery mildew tolerance with molecular sizes of 1100 bp in genotype number 7, which exhibited moderate tolerance to powdery mildew, and another positive marker in genotype 5 with a molecular size of 600 bp. Five markers were generated by the primer SCoT-25, one positive with a molecular size of 1000 bp detected in genotype number 2, three negative markers with sizes of 800, 600, and 480 bp presented in genotypes 14, 16, and 2, and one negative marker in genotype 5 with a molecular size of 700 bp, which may be considered a negative marker for powdery mildew tolerance.

For primer SCoT-1, one specific marker was generated in this regard: one positive marker for genotype 5, which exhibited moderate tolerance to powdery mildew with a molecular size of 1300 bp. In addition, with the primer SCoT-9, one negative marker was observed with a molecular size of 99 bp in genotype 1. The primer SCoT-12 exhibited two negative-specific markers with a molecular size of 800 and 300 bp in genotypes 8 and 10. The primer SCoT-13 exhibited two negative-specific markers for genotype 2 with a molecular size of 900 and 400 bp. One positive and one negative specific marker were generated by the primer SCoT-34 in genotypes 15 and 18 with a molecular size of 1500 and 400 bp, respectively. Also, one positive specific marker with a molecular size of 950 bp was generated by the primer SCoT-35 for genotype 1. One negative-specific marker was generated by the primer SCoT-36 in genotype 8 with a molecular size of 400 bp.

#### ISSR analysis

**Fig. 4:**
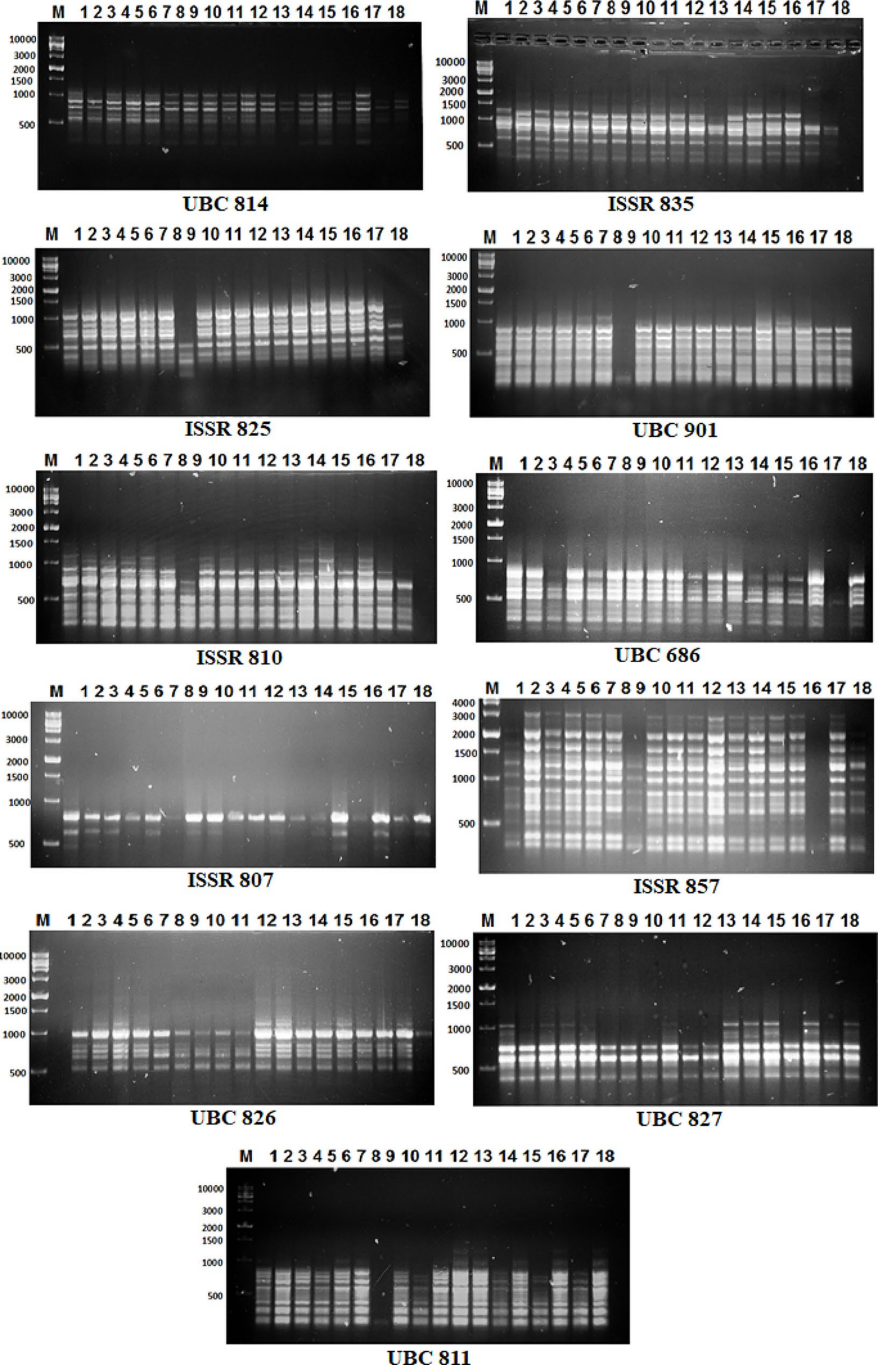
ISSR fingerprints of the ten barely genotypes tested using 11 primers. M (marker), Lanes 1–18 flax genotypes. Stewart (1), Polk (2), Birio (3), Kenya (4), Akmolinsk ACC (5), Abyssinian Brown (6), Leona (7), Willaton Brown (8), Victory (9), Bison (10), B. Golden Sel (11), Barnes (12), Bisbee (13), Pale Blue Crimped (14), Burk (15), Ward (16), B6 x Kugler C (17), Linora (18)

The level of polymorphism and a comparison of the discriminating capacity of ISSR markers are summarized in Table 8. Using 11 ISSR primers, a total of 97 bands (76 polymorphic and 21 monomorphic) were observed, with 78.4% (polymorphism), including 26 unique bands (3 positive specific markers and 23 negative specific markers). Their molecular size ranged from 100 to 1000 bp (Fig. 4; Table 8). The ISSR-857 and UBC901 primers recorded the highest polymorphism values (94.1% and 90.0%). The lowest polymorphism percentage was observed in primer ISSR-810 (40.0%). The ISSR-857 primer produced the highest number of polymorphic bands (16).

**Table 8:** Bands variation and polymorphism percentage for flax genotypes using ISSR primers

Primer	No. of bands	Polymorphic bands	Monomorphic bands	Polymorphism %	PIC	Unique bands	Ve + U.B	Ve- U.B	Genotypes	Band size (bp)
ISSR primers										
UBC814	8	6	2	75.0%	0.919	2	0	2	12	600, 550
ISSR-835	8	6	2	75.0%	0.914	2	1	1	14, 18	950, 600
ISSR-825	11	9	2	81.8%	0.942	3	1	2	7	1000, 700, 100
UBC901	10	9	1	90.0%	0.935	4	0	4	7	800, 700, 600, 400
ISSR-810	10	4	6	40.0%	0.924	1	0	1	7	900
UBC686	8	7	1	87.5%	0.906	3	0	3	17	800, 600, 200
ISSR-807	3	2	1	66.7%	0.851	0	0	0	-	-
ISSR-857	17	16	1	94.1%	0.971	5	1	4	16, 6, 16, 16, 16	1200, 1100, 1000, 900, 700
UBC826	6	5	1	83.3%	0.878	3	0	3	18	700, 600, 500
UBC827	7	4	3	57.1%	0.922	0	0	0	-	-
UBC811	9	8	1	88.9%	0.933	3	0	3	7	700, 600, 200
**Total**	**97**	**76**	**21**	**-**	**-**	**26**	**3**	**23**	**-**	**-**
**Average**	**-**	**-**	**-**	**78.4%**	**0.918**	**-**	**-**	**-**	**-**	**-**

Twenty-six amplified bands (3 positive and 823 negative) were derived from ISSR primers (Table 8). The ISSR-857 primer revealed the highest number of unique bands (1 positive and 4 negative markers) in genotypes 6 and 16 with molecular sizes of 1200, 1100, 1000, 900, and 700 bp. Also, the UBC901 primer exhibited four unique bands (negative markers) with molecular sizes of 800, 700, 600, and 400 bp in genotype 7, which considered moderate tolerance to powdery mildew. ISSR-825 detected one positive and two negative unique markers in genotype 7 with molecular sizes of 1000, 700, and 100 bp. Also, three negative unique markers were detected by UBC811 in genotype 7 with molecular sizes of 700, 600, and 200 bp. Two markers were generated by primer ISSR-835, one positive with a molecular size of 950 bp detected in genotype number 14, which is considered sensitive to powdery mildew, and one negative marker with a molecular size of 600 bp presented in genotype 18, which is considered sensitive to powdery mildew. Also, the UBC826 primer generated three negative markers in genotype 18, which are considered sensitive to powdery mildew with molecular sizes of 700, 600, and 500 bp. In addition, the ISSR-810 primer generated one negative marker related to powdery mildew tolerance in genotype number 7 with a molecular weight of 900 bp. Three negative markers were generated by the UBC686 primer in genotype 17, which were considered moderately sensitive to powdery mildew with molecular sizes of 800, 600, and 200 bp.

The findings demonstrated that, by accounting for the total number of alleles and their relative frequencies, the PIC value assesses a locus’s discriminatory potential. PIC values vary from 0 (monomorphic) to 1 (very highly discriminative, with many alleles at equal frequencies) (Tables 7 and 8). The SCoT primers’ polymorphism information content (PIC) varied from 0.850 to 0.958, averaging 0.926. SCoT-12 exhibited the highest PIC, measuring 0.958, whilst SCoT-22 displayed the lowest PIC, measuring 0.850. The ISSR primers’ polymorphism information content (PIC) varied from 0.851 to 0.971, with an average of 0.918. The PIC value of 0.971 in ISSR-857 was found to be the highest, and 0.851 in ISSR-807 was the lowest.

#### Cluster analysis

**Fig. 5:**
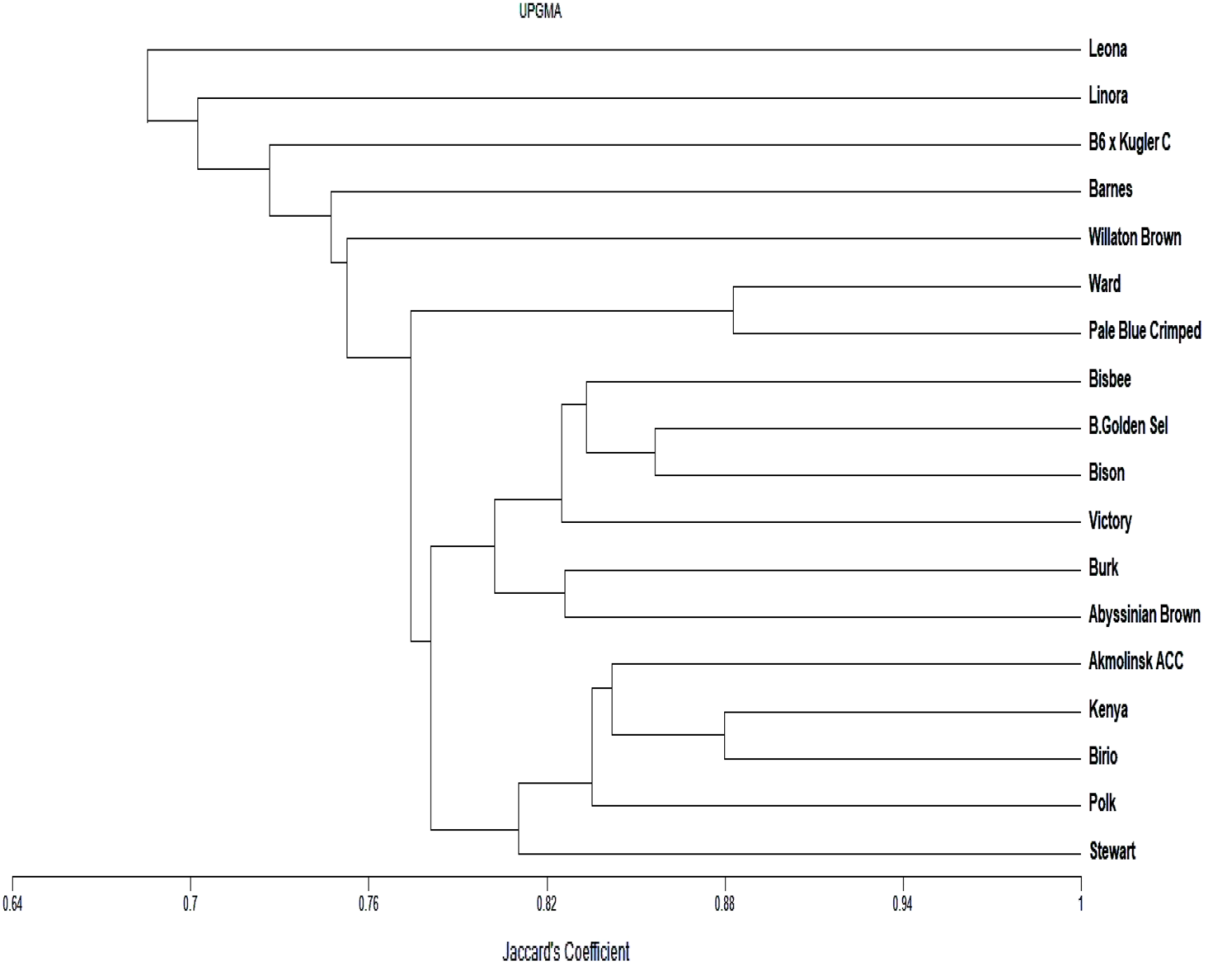
UPGMA cluster analysis based on Jaccard similarity coefficient, showing the genetic relationships among the ten barely genotypes tested, obtained from SCoT, and ISSR markers

The dendrogram of the 18 genotypes that were studied is displayed in Fig. 5 and is based on the SCOT and ISSR markers. The genotypes fell into two major clusters, according to UPGMA cluster analysis. Leona (genotype 7), which was thought to have a distinct phenetic line and considerable resistance to powdery mildew, was a part of the first main cluster. The remaining 17 genotypes are clustered into a sub-cluster within the second main cluster. Thus, this supported their high degree of commonality.

Table 9 shows the levels of similarity matrix among the 18 genotypes that were examined using the SCoT and ISSR. The Pale Blue Crimped (genotype 14) and Ward (genotype 16) genotypes have the highest similarity (88.3%) in the degree of similarity matrix between the genotypes under study; both genotypes are susceptible to powdery mildew infection. Leona (genotype 7), which is considered moderately resistant to powdery mildew, and Barnes (genotype 12), which is considered highly susceptible, have the lowest similarity between them (60.1%).

**Table 9:** Similarity matrix values generated using NTSYS software for the data produced from SCoT and ISSR primers for the 18 flax genotypes

Genotype number		1	2	3	4	5	6	7	8	9	10	11	12	13	14	15	16	17	18
	Genotype name	**Stewart**	**Polk**	**Birio**	**Kenya**	**Akmolinsk ACC**	**Abyssinian Brown**	**Leona**	**Willaton Brown**	**Victory**	**Bison**	**B.Golden Sel**	**Barnes**	**Bisbee**	**Pale Blue Crimped**	**Burk**	**Ward**	**B6 x Kugler C**	**Linora**
1	Stewart	1.000																	
2	Polk	0.804	1.000																
3	Birio	0.790	0.836	1.000															
4	Kenya	0.815	0.842	0.880	1.000														
5	Akmolinsk ACC	0.832	0.828	0.824	0.859	1.000													
6	Abyssinian Brown	0.786	0.774	0.798	0.823	0.830	1.000												
7	Leona	0.699	0.679	0.672	0.706	0.707	0.795	1.000											
8	Willaton Brown	0.726	0.742	0.727	0.761	0.742	0.761	0.652	1.000										
9	Victory	0.741	0.775	0.770	0.816	0.775	0.786	0.697	0.814	1.000									
10	Bison	0.709	0.770	0.774	0.799	0.769	0.789	0.683	0.777	0.852	1.000								
11	B.Golden Sel	0.730	0.791	0.786	0.782	0.790	0.811	0.686	0.789	0.813	0.856	1.000							
12	Barnes	0.726	0.751	0.737	0.733	0.742	0.742	**0.610**	0.737	0.715	0.747	0.788	1.000						
13	Bisbee	0.772	0.778	0.774	0.807	0.814	0.798	0.694	0.776	0.810	0.823	0.844	0.775	1.000					
14	Pale Blue Crimped	0.804	0.783	0.752	0.784	0.827	0.784	0.694	0.727	0.741	0.762	0.809	0.772	0.824	1.000				
15	Burk	0.744	0.768	0.782	0.797	0.804	0.826	0.710	0.755	0.770	0.802	0.843	0.755	0.820	0.832	1.000			
16	Ward	0.794	0.746	0.715	0.764	0.790	0.755	0.698	0.689	0.704	0.734	0.763	0.742	0.787	**0.883**	0.777	1.000		
17	B6 × Kugler C	0.667	0.719	0.761	0.718	0.684	0.747	0.627	0.684	0.719	0.773	0.785	0.733	0.753	0.706	0.791	0.658	1.000	
18	Linora	0.670	0.677	0.689	0.685	0.688	0.713	0.640	0.678	0.704	0.728	0.721	0.747	0.728	0.692	0.708	0.688	0.722	1.000

#### Principal component analysis (PCA)

**Fig. 6:**
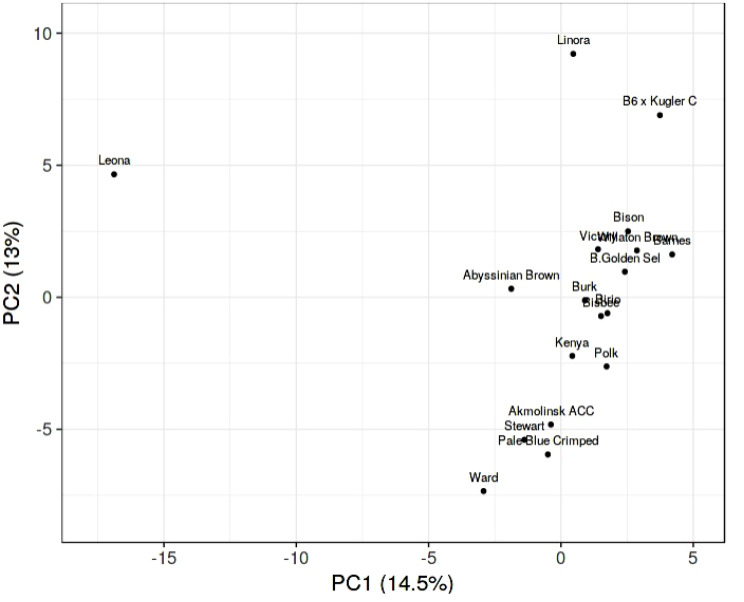
An illustration of the genetic diversity expressed in 18 Egyptian barley genotypes, according to a principal component analysis (PCA) based on polymorphism of SCoT and ISSR markers, using PAST software

The genetic diversity parameter data from the SCoT and ISSR markers, multivariate clustering, and PCA analysis were used to evaluate the genetic diversity of the genotypes under study. The SCoT and ISSR markers are reliable in identifying the genotypes under test in a PCA scatter plot. The two PCAs showed 13% (PCA2) and 14.5% (PCA1). It separated Leona (genotype 7), which is considered moderate resistance to powdery mildew disease, in a separated group from all genotypes, while the other genotypes were found in one group and were close to each other (Fig. 6).

#### Multivariate Heatmap

**Fig. 7:**
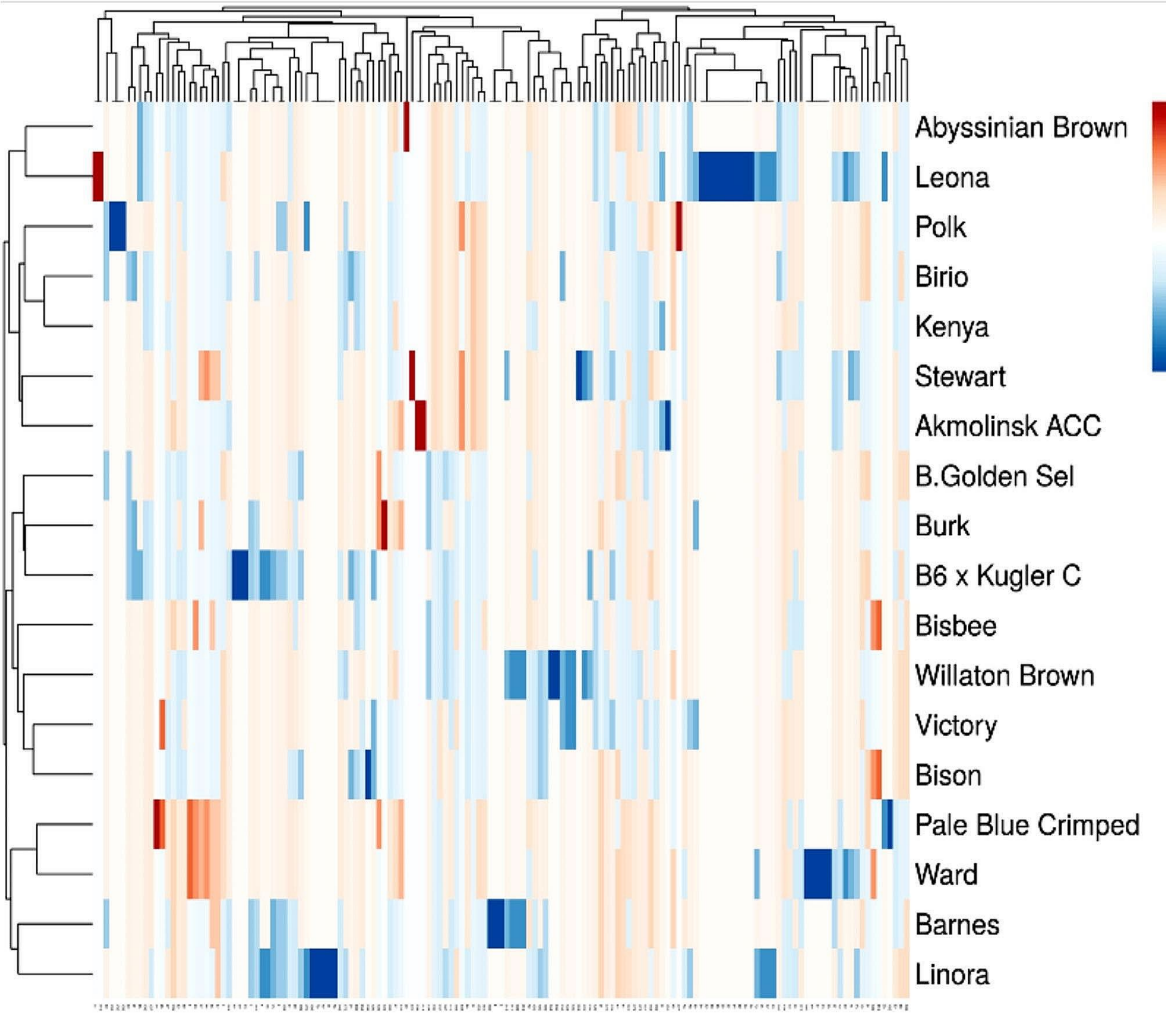
Multivariate Heatmap illustrating the genetic diversity of 18 flax genotypes, based on 12 SCoT primers and 11 ISSR primers for using the module of a Heatmap of ClustVis—an online tool for clustering and visualizing of multivariate data

Plant breed genetic variation is depicted in detail in a heatmap, and additional information about this genetic variance is usually obtained by multivariate compound similarity analysis. Heatmap, produced by ClustVis, is an online application for grouping and displaying compound similarities in multivariate data. The columns display the five clusters that were created from the genotypes of eighteen flax plants (Fig. 7). The first cluster was divided into two sub-clusters: the Linora (genotype number 18) and Barnes (genotype 12), which were all part of the first sub-cluster. The genotypes Pale Blue Crimped (genotype 14) and Ward (genotype 16) were distinguished as part of the two sub-clusters. Bison (genotype 10), Victory (genotype 9), Willaton Brown (genotype 8), Bisbee (genotype 13), B6 x Kugler C (genotype 17), Burk (genotype 15), and B. Golden Sel (genotype 11) made up the second cluster. In addition, Akmolinsk ACC (genotype 5), Stewart (genotype 1), Kenya (genotype number 4), Birio (genotype 3), and Polk (genotype 2) were the third cluster. Abyssinian Brown (genotype 6) was in the fourth cluster, and Leona (genotype 7) made up the fifth cluster (Fig. 7).

## Discussion

In the northern governorates of the Nile Delta, flax is produced for its seeds and fibers. Warm rainy weather is typical in this location in the latter part of the flax-growing season. When pathogenic isolates of the fungus cause powdery mildew (PM), the weather is conducive to the epiphytotic spread of powdery mildew. This disease has become more significant during the past two decades, most likely as a result of the emergence and quick spread of novel races that may now target previously resistant genotypes [4].

In the present study, the genotypes showed considerable variation in powdery mildew severity, ranging from 18.10% on Leona to 92.03% on Polk. These findings are consistent with a two-year field study undertaken by Mohamed et al. [1] to assess the severity of powdery mildew on nine different genotypes of flax. They discovered a significant range in powdery mildew severity, from 8.1 on the Ottowa 770 B genotype to 97% on the Cortland genotype. Hussein and Omar [39] assessed 12 flax genotypes’ responses to powdery mildew in a two-year field investigation. Five of the genotypes were exotic, and seven were indigenous. Furthermore, Aly et al. [4] assessed the severity of powdery mildew (PM) on 18 flax genotypes over a year. Two separate groups were created from the tested genotypes. There were twelve highly resistant genotypes in the initial cluster. On these genotypes, the disease severity varied from 1 to 10%. The second cluster included six highly sensitive genotypes with disease severity ranging from 90 to 100%. The indigenous genotypes were often more resistant to the disease, most likely due to their adaptation to the *Oidium lini* population in the area.

Cultivating resistant genotypes is the best way to manage the disease and significantly lower the occurrence of powdery mildew. In this study, 18 genotypes were assessed for disease resistance to choose materials that may be used in disease-integrated management programs and/or flax breeding programs. It is commonly acknowledged that the safest, most practical, and most efficient way to safeguard crops against disease is through the use of resistant genotypes [40].

Molecular and biochemical markers provide methods to reveal the genetic diversity among genotypes based on polypeptide chains and nucleic acid polymorphisms. Evaluating the genetic variability within a cultivated crop has significant implications for plant breeding and the conservation of genetic resources [40]. The present study evaluated the level and pattern of genetic diversity in 18 flax genotypes using biochemical markers, protein profiles, and two molecular maker systems, SCoT and ISSR markers. The moderately resistant genotype 7 (Leona) exhibited considerably greater levels of antioxidant enzymes (PPO, POD, CAT, SOD, and PAL) in comparison to the other genotypes. These outcomes are consistent with Chen et al. [41], who discovered that, following infection, two flax genotypes—one resistant to pasmo and the other susceptible—showed varying levels of defense enzyme activity and malondialdehyde (MDA) content in their leaves. The outcomes demonstrated that the superoxide dismutase activity in the resistant genotypes was considerably higher than susceptible genotypes in the early phases of pathogen infection. Furthermore, a good correlation was found between the variations in the resistance of different flax genotypes to pasmo and the alterations in peroxidase, catalase, and polyphenol oxidase activities in the infected leaves.

The most common defense enzymes are POD, CAT, PAL, SOD, and PPO. These enzymes can directly inhibit and kill pathogens during a plant-pathogen interaction, enabling plants to resist disease. They can also contribute to creating disease-resistant secondary compounds or metabolizing reactive oxygen species in plants [9]. CAT is an enzyme that is found in many different types of organisms. It mostly scavenges the hydrogen peroxide generated during plant metabolism, which helps to protect plants [10]. Flax leaves from resistant and susceptible genotypes significantly boosted CAT activity [42]. CAT activity showed significantly more noticeable increases in the tolerant genotypes than in the susceptible genotypes. Quickly eliminating H_2_O_2_ may boost catalase activity and shield it from oxidative stress.

Among the essential enzymes in the plant’s enzymatic defense system is POD. It takes part in both quality control and lignin synthesis. It can increase mechanical strength, lessen susceptibility to extracellular enzyme breakdown, stop pathogen invasion, trigger phenol oxidation, and encourage browning of plants [10]. One of the primary antioxidant enzymes that scavenge free radicals in plants that contain superoxide anion (SOD) can stop cell membrane peroxidation [43]. PPO is a common component of both plants and animals. It can catalyse the conversion of polyphenols to quinones, which can impede and kill pathogens [44]. POD and PPO play a crucial part in the defense mechanism against infections by oxidizing phenolic chemicals into quinines, which boosts the antibacterial action of the compounds. As a result, it might directly impact halting the growth of the pathogen, hastening the death of cells at the infection site, blocking the spread of infection, or creating a toxic environment that prevents the pathogen from growing inside the cells [42].

PAL is regarded as a crucial physiological indicator of plant resilience. Numerous phenols, flavonoids, terpenes, and other compounds can be produced via the PAL-regulated phenylpropane metabolic pathway. It is crucial for plant development and growth, disease resistance, insect pest control, and other factors [45].

The moderate resistant genotypes (Leona) showed a decreased MDA and H_2_O_2_ content in their shoots compared to the other genotypes. These findings are consistent with those of Mohamed et al. (2012) [1], who discovered that MDA was the only factor positively correlated with powdery mildew severity. This finding could imply that lipid peroxidation following an infection is a significant factor in determining flax’s sensitivity to powdery mildew. Elevated MDA production in particularly susceptible genotypes suggests significant levels of post-infectional lipid peroxidation, which may increase the permeability of the flax membrane and worsen the disease.

Strong oxidants like MDA can lower the cell membrane system’s electric resistance and membrane fluidity, ultimately destroying the membrane’s structural and physiological integrity. It is strongly correlated with the extent of cell membrane damage since MDA is so hazardous to cells that it can disrupt cell membrane function and harm a variety of important molecules [41]. Consequently, damage to plant cells is directly caused by increased MDA levels. To some extent, MDA, the end product of membrane lipid peroxidation, represents plants’ membrane peroxidation level. MDA and plant disease resistance are so strongly associated [41].

The current study’s findings demonstrated that the moderately resistant genotype (Leona) shoots had a greater concentration of phenolic and flavonoids than the other genotypes. Phenolic-based defense responses in resistant plants are typified by the early and quick deposition of phenolics at the infection site, eliminating the pathogen. Numerous research findings indicate that phenols esterify to cell wall components and that phenols accumulate and deposit in and on cell walls. These processes are typically interpreted as increasing resistance to fungal hydrolytic enzymes and acting as a physical barrier against fungal penetration [42]. It is commonly recognized that most phenolics have fungi-toxic effects mostly due to their interaction with lipids or phospholipids, which increases the permeability of fungal membranes, allows cell contents to leak out, and causes cytoplasm to aggregate [2]. Therefore, elevated phenolic compound levels in leaf tissues may improve flax resistance to powdery mildew infection [2].

Our study compared the SDS-PAGE proteins of different flax genotypes under biotic stress. The SDS-PAGE protein profile of total soluble protein from flax genotypes showed different band patterns between genotypes. In several genotypes of flax, protein bands disappeared due to powdery mildew infection. According to earlier research by Mahmoud and Abd El-Fatah [40], the reason for the absence of these protein bands in the infected genotypes is due to the blockage of the trigger for resistant gene transcription, which results in the production of disease-related proteins.

However, some new protein bands were created in infected genotypes, serving as protein markers for resistance mechanisms that make plants more resilient to pathogens. With their adaptable sensing systems, plants probably use many signal transduction pathways and recognition systems to initiate defense mechanisms. It has been discovered that various protein types have distinct functions in the plant defense system and resistance to plant diseases [40].

The high degree of polymorphism suggests that the genotypes under study are genetically divergent, indicating that these marker systems were perfect for examining the genetic diversity among closely related genotypes. The efficacy of SCoT and ISSR as genetic markers was contrasted to assess the genetic diversity of flax genotypes. Our findings generally concur with those of a few earlier research that show the importance of molecular markers in distinguishing between susceptible and resistant genotypes. Singh et al. [46] and Satya and Chakrabort [47] employed ISSR, RAPD, and SSR analysis to examine the genetic diversity of flax and mung bean. Poolswat et al. [48] discovered that the ISSR marker is a very effective method for mapping the mung bean powdery mildew resistance gene. Also, Osman [15] discovered that ISSR primers effectively distinguish flax genotype-resistant and susceptible genotypes from powdery mildew resistance. According to the ISSR analysis, there was a lot of genotype polymorphism.

In this study, 12 SCoT primers produced a total of 119 bands; 48 bands were monomorphic, and 71 bands were polymorphic, with 59.7% (polymorphism), including 18 unique bands (6 positive specific markers and 12 negative specific markers). While 11 ISSR primers produced a total of 97 bands (76 polymorphic and 21 monomorphic), 78.4% were polymorphic, including 26 unique bands (3 positive specific markers and 23 negative specific markers). These outcomes are consistent with Osman et al. [19], who evaluated 12 elite flax genotypes using the thirteen primers for each of the two SCoT and ISSR tests, yielding a total of 209 and 177 bands, respectively. In addition, Ahmed et al. [49] employed 10 ISSR primers to analyze the genetic diversity within nine flax genotypes, 88 DNA fragments were detected with an average of 8.8 bands per primer and a polymorphism average of 54.8%. Kumari et al. [18] examined 28 flax genotypes using 11 ISSR primers, finding 58 bands with an average of 5.2 per primer. To distinguish between the 18 genotypes of flax, the two molecular markers under investigation were able to produce distinct fingerprints that might be helpful in plant breeding programs. These findings are consistent with those of Ahmed et al. [49], Ahmed et al. [50], and Osman et al. [19].

Based on the SCOT and ISSR markers, the dendrogram of the 18 genotypes in this study showed that they were divided into two major clusters. These results are similar to Pali and Mehta [51], who used similarity index data from SSR and ISSR markers, where 48 flax genotypes were sorted into two main groups. Also, by using ISSR marker data, a two-grouped dendrogram of nine flax genotypes was produced [49]. In addition, Osman et al. [19] discovered that the twelve flax genotypes were split into two major groups by the dendrogram of genetic relationships created using SCoT and ISSRs data.

The first evidence of the association between disease and diversity appeared in reaction to the destruction caused by epidemics in agricultural areas. As a result, the agricultural community has decades of real-world experience with the advantages and difficulties of using genetic variety as a disease management strategy. Variety is of relevance because it can protect important resistance genes against parasite counter-adaptation (“durable resistance”) and increase crop production during a growing season [52].

## Conclusion

The present study reports the genetic diversity of 18 flax genotypes for resistance or susceptibility to powdery mildew. These genotypes were broadly grouped into four groups, i.e., highly susceptible, susceptible, moderately susceptible, and moderately resistant. The present study revealed the feasibility of prescreened SCoT, ISSR, proteins, and biochemical markers (antioxidant enzymes, phenolics, and flavonoids) for genetic diversity analysis and their potential association with powdery mildew resistance. In the current study, two distinct molecular marker types—SCoT and ISSR—were employed to create unique molecular markers that could be used in genotype identification and to create a distinct fingerprint for every genotype of flax. Moreover, the high polymorphism observed highlights the allelic richness of the analyzed genotypes, which is useful for the crop improvement program. Leona (genotype 7), which is considered moderately resistant to powdery mildew, may be used in the breeding program for flax. This manuscript helps breeders use resistant and moderate genotypes in their breeding programmes to increase the resistance of flax genotypes to powdery mildew and decrease yield losses. In addition to using these resistance genotypes to do crosses between genotypes. Diversity in plant genetic resources (PGR) provides opportunity for plant breeders to develop new and improved genotypes with desirable characteristics, which include both farmer-preferred traits (yield potential and large seed) and breeders preferred traits (pest and disease resistance).

### Electronic supplementary material

Below is the link to the electronic supplementary material.

Supplementary Material 1

## Data Availability

Data is provided within the manuscript or supplementary information files.
