# Supplementary material for: Investigation of genetic diversity using molecular and biochemical markers associated with powdery mildew resistance in different flax (Linum usitatissimum L.) genotypes
Source: BMC Plant Biol. 2024 May 17;24:412. doi: 10.1186/s12870-024-05113-5 (PMC11100107; doi:10.1186/s12870-024-05113-5)
Supplement: Supplementary file 1 — Supplementary Material 1 [file 12870_2024_5113_MOESM1_ESM.docx]

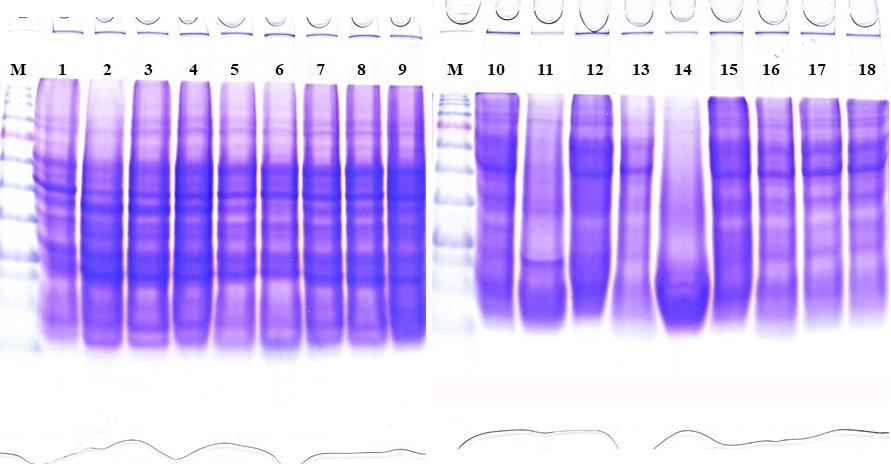


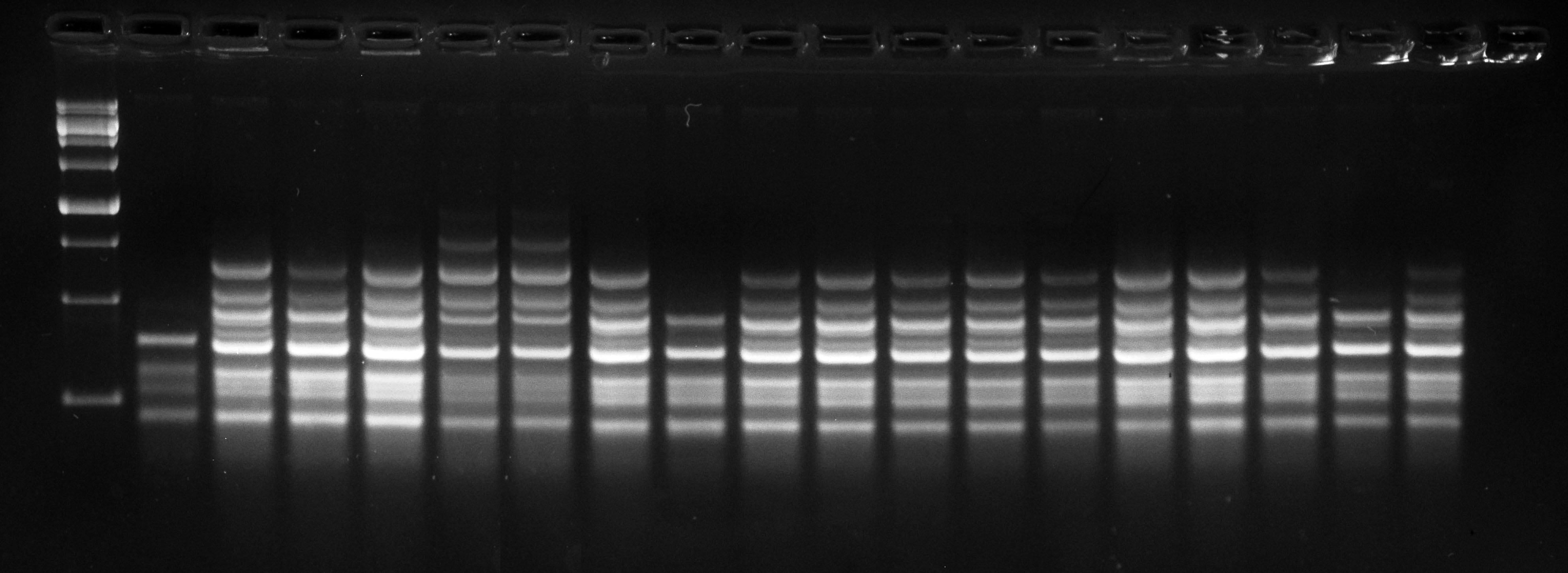


SCoT 9


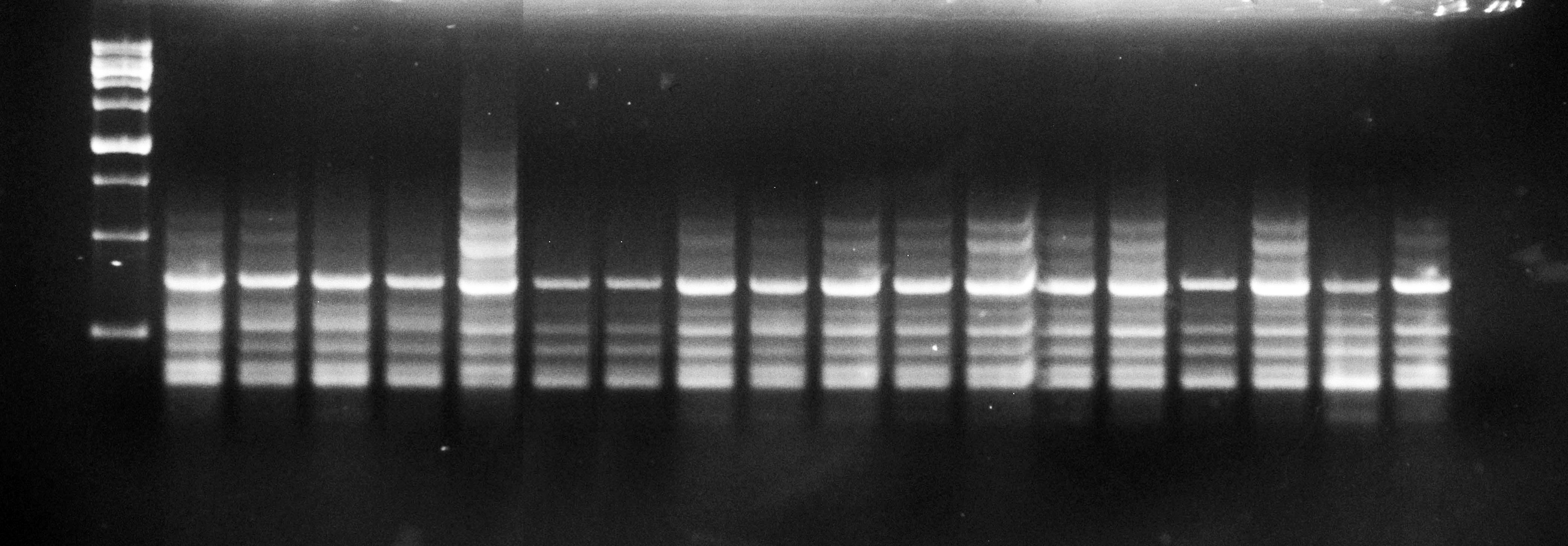


SCoT 1


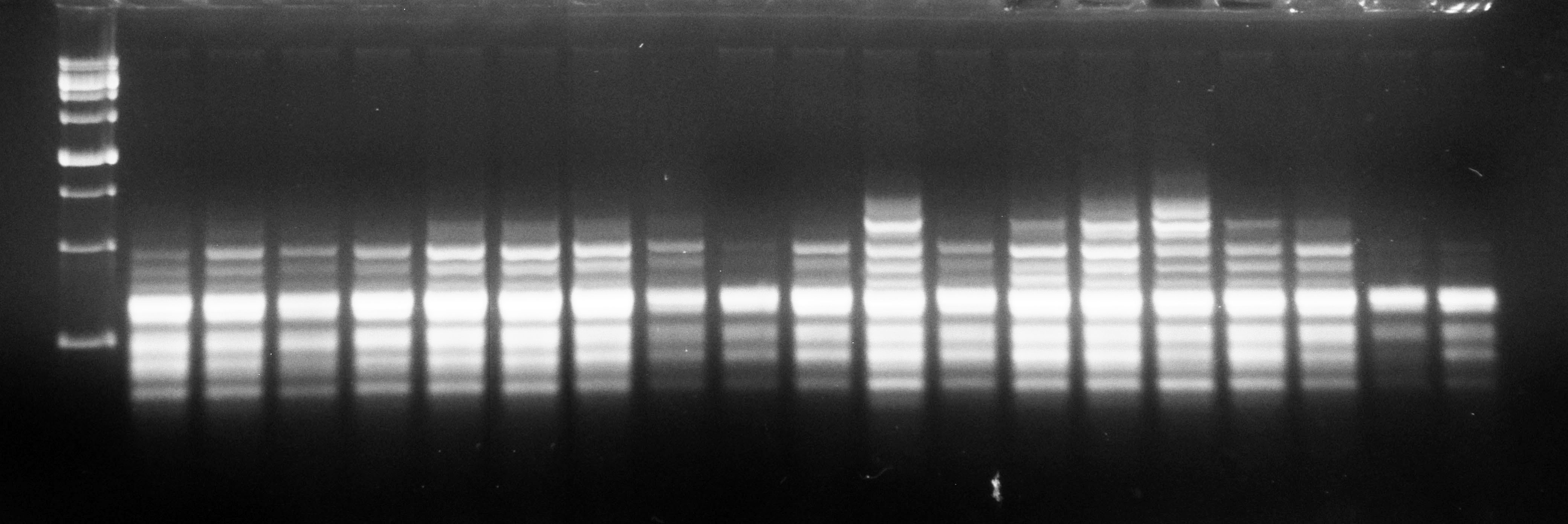
SCoT 34


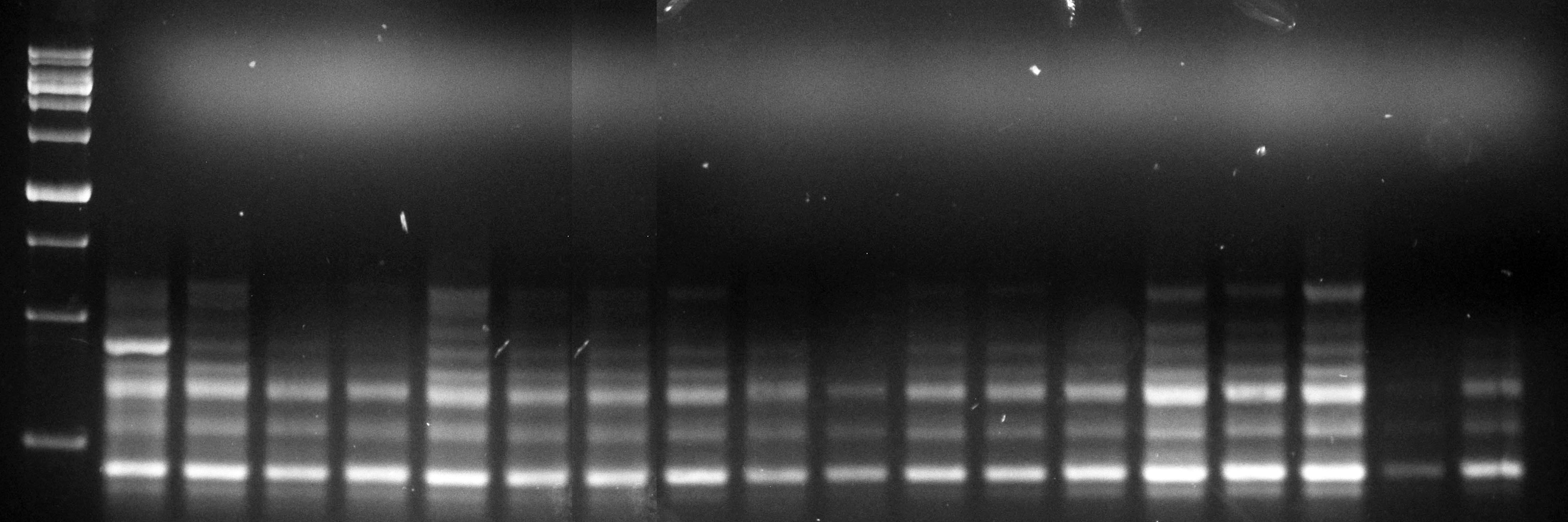


SCoT 35


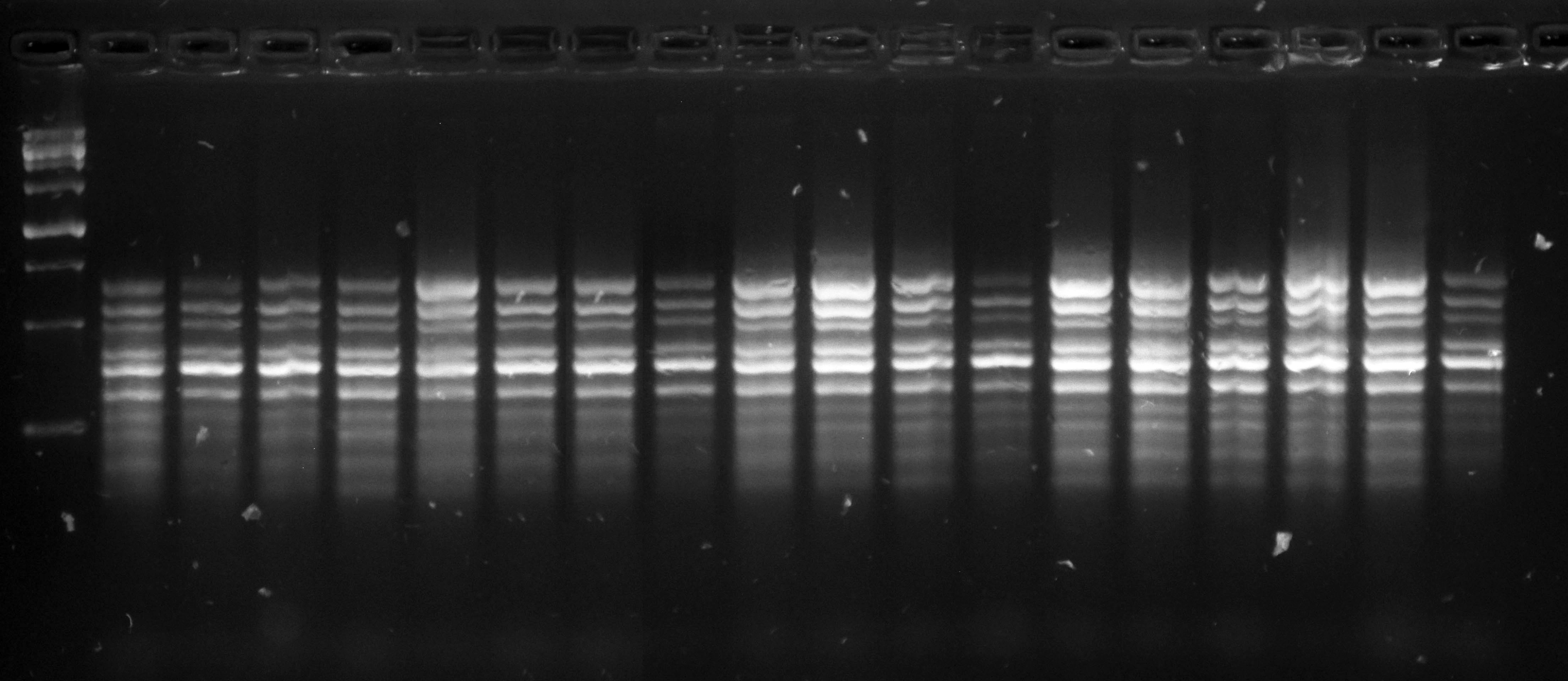


SCoT 36


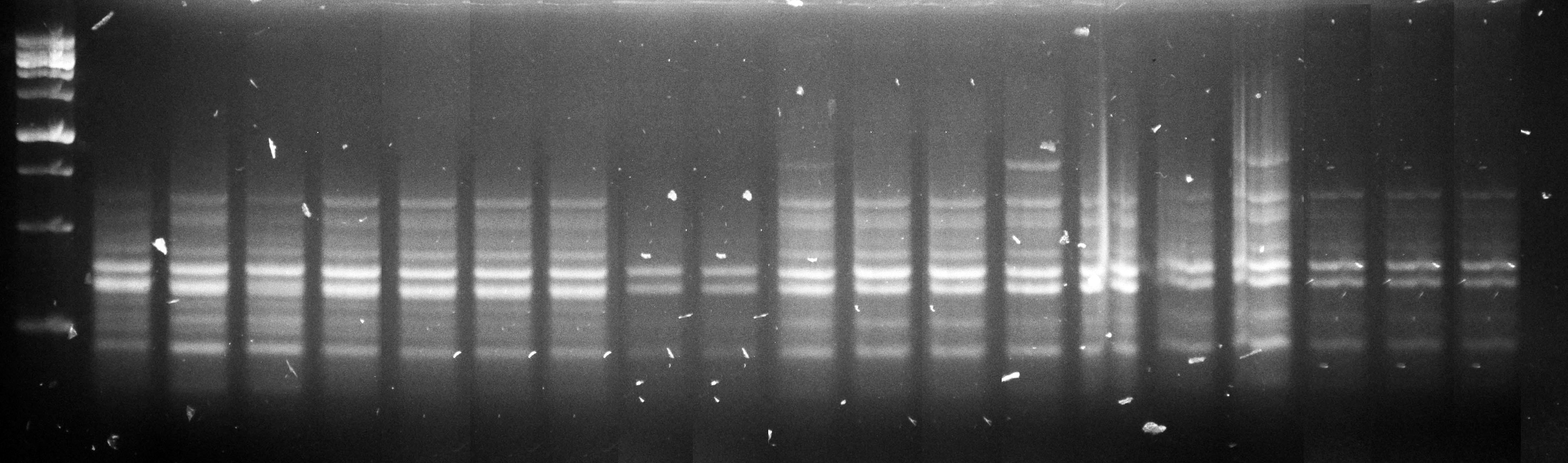


SCoT 11


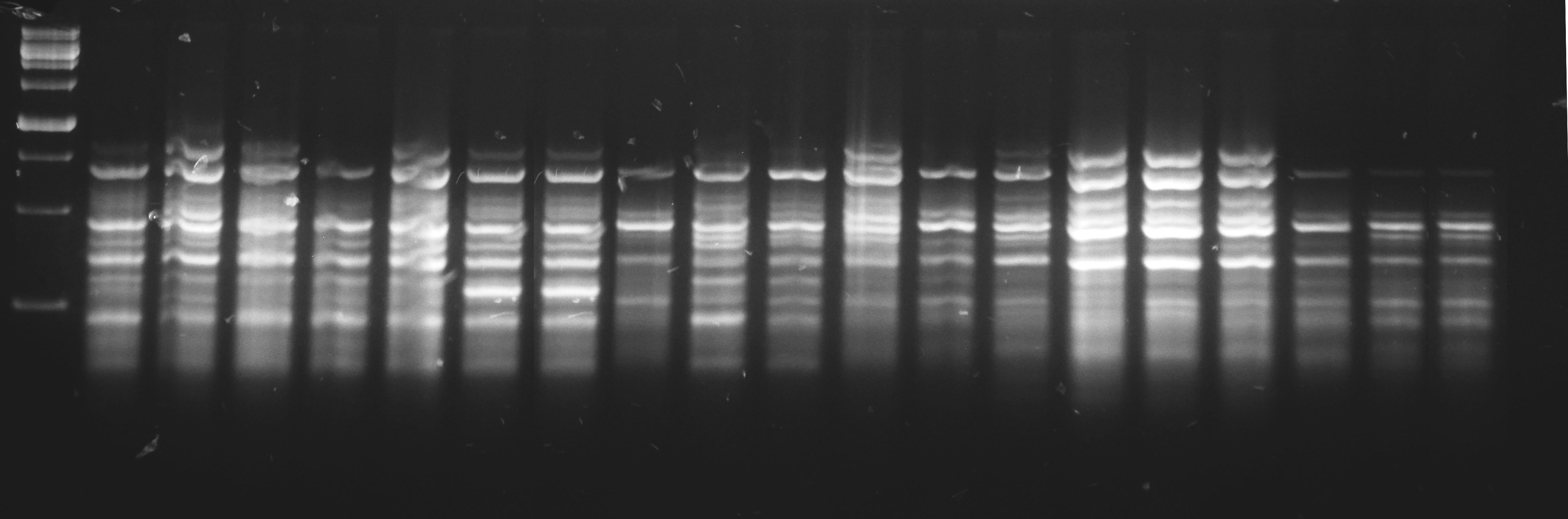


SCoT 12


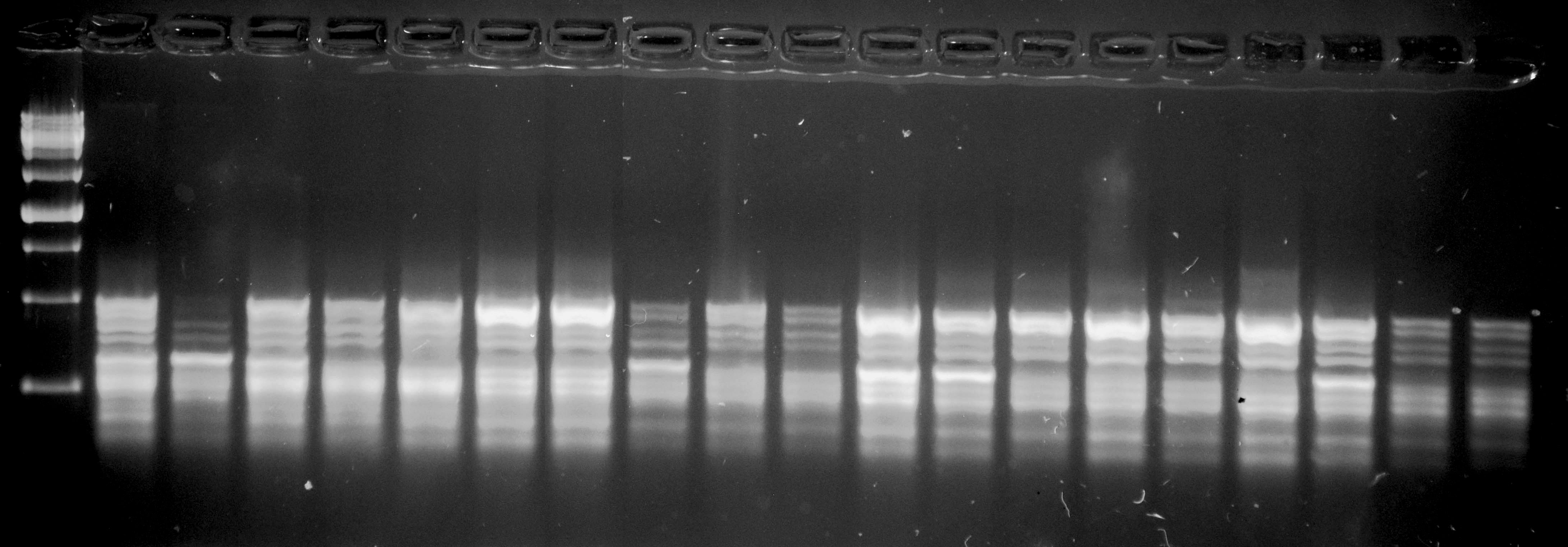


SCoT 13


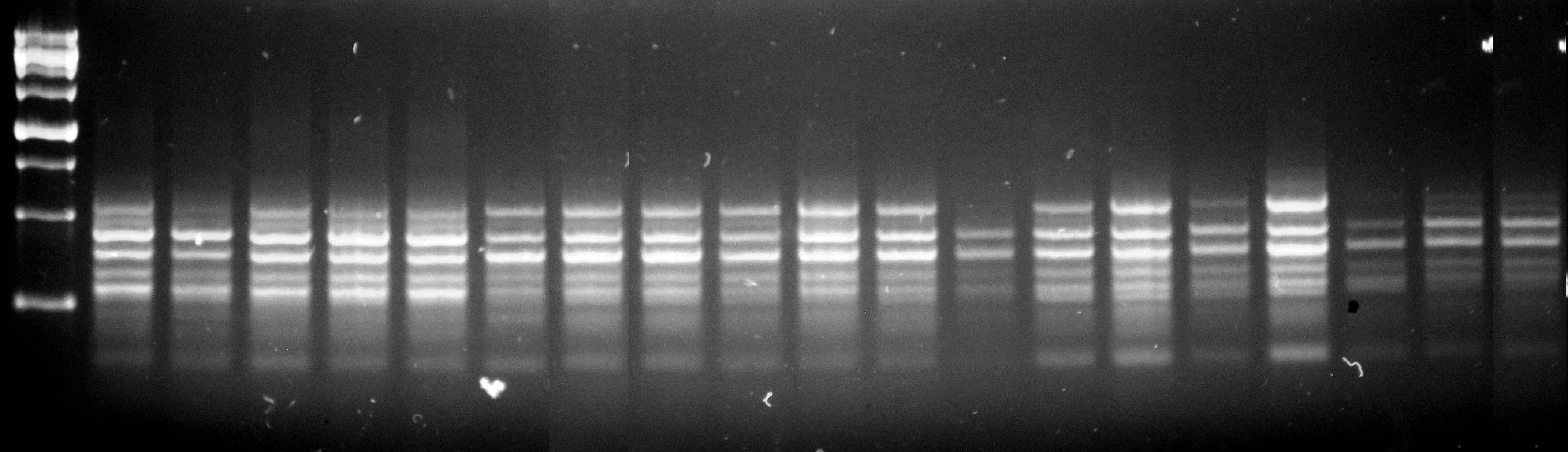


SCoT 21


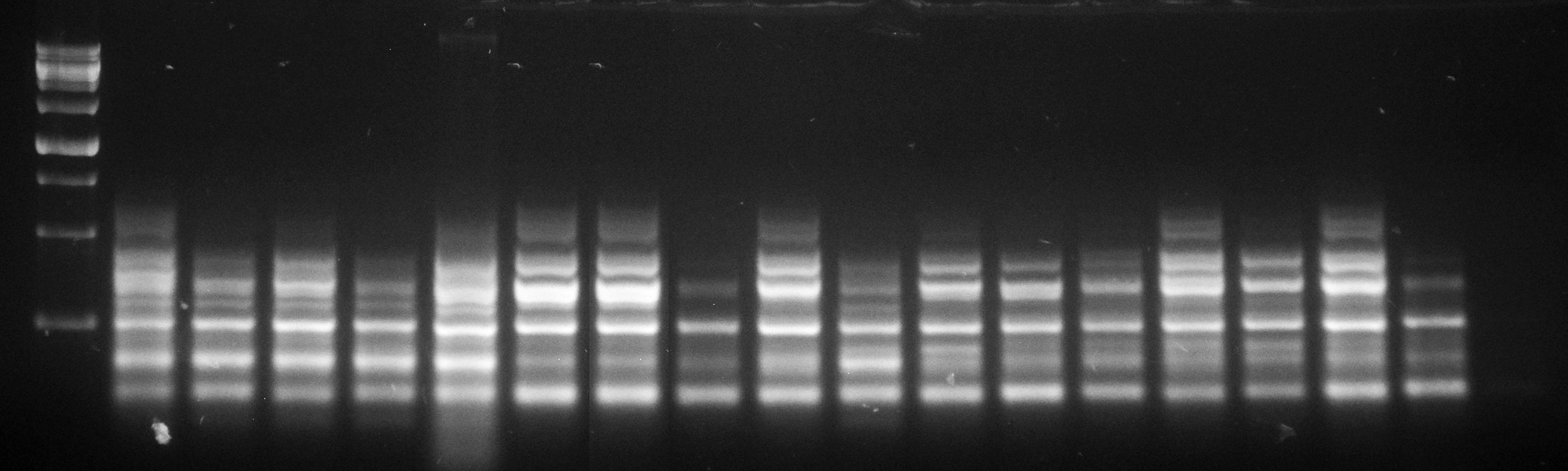


SCoT 22


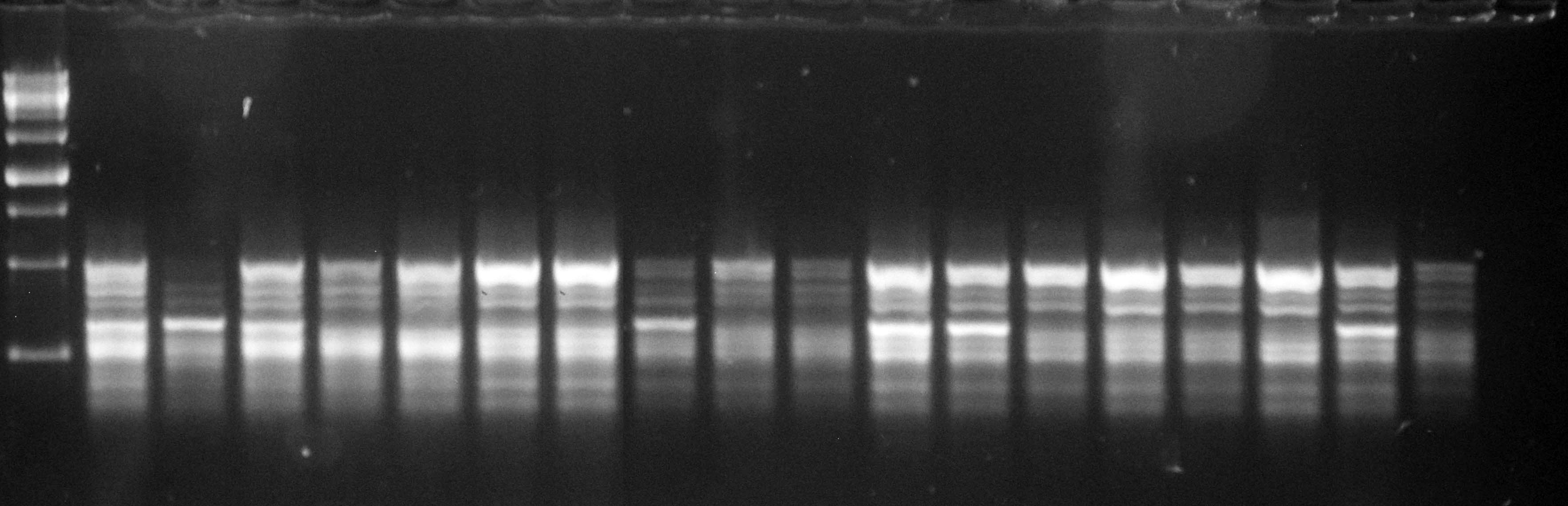


SCoT 25


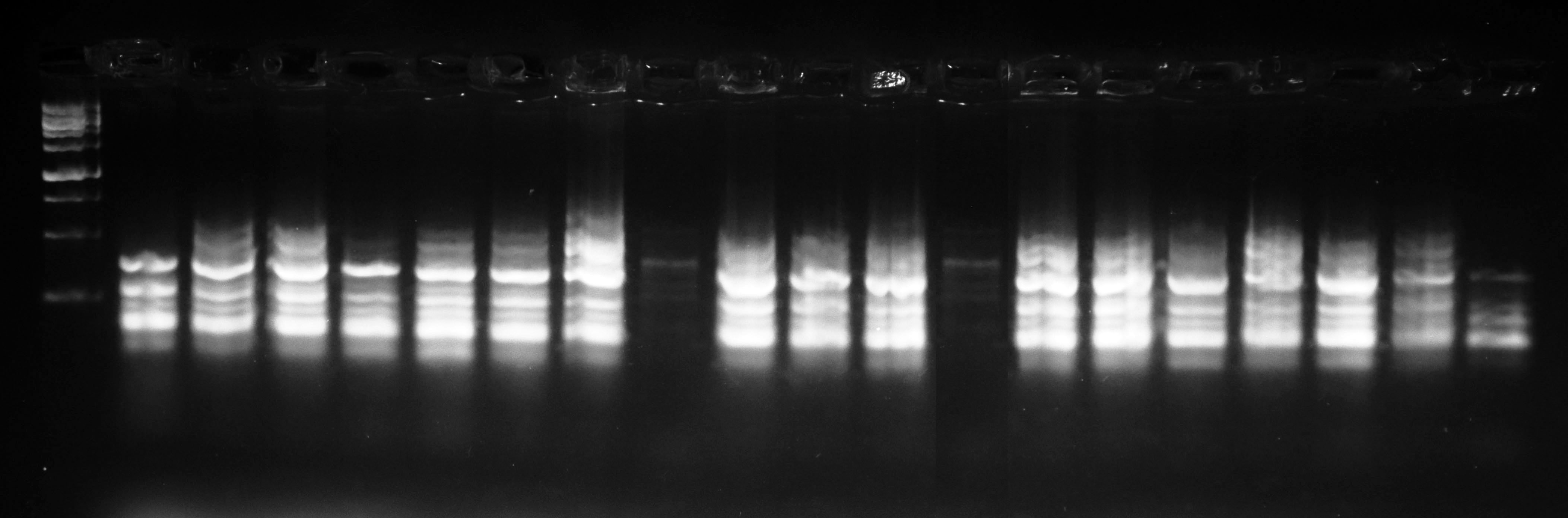


SCoT 6


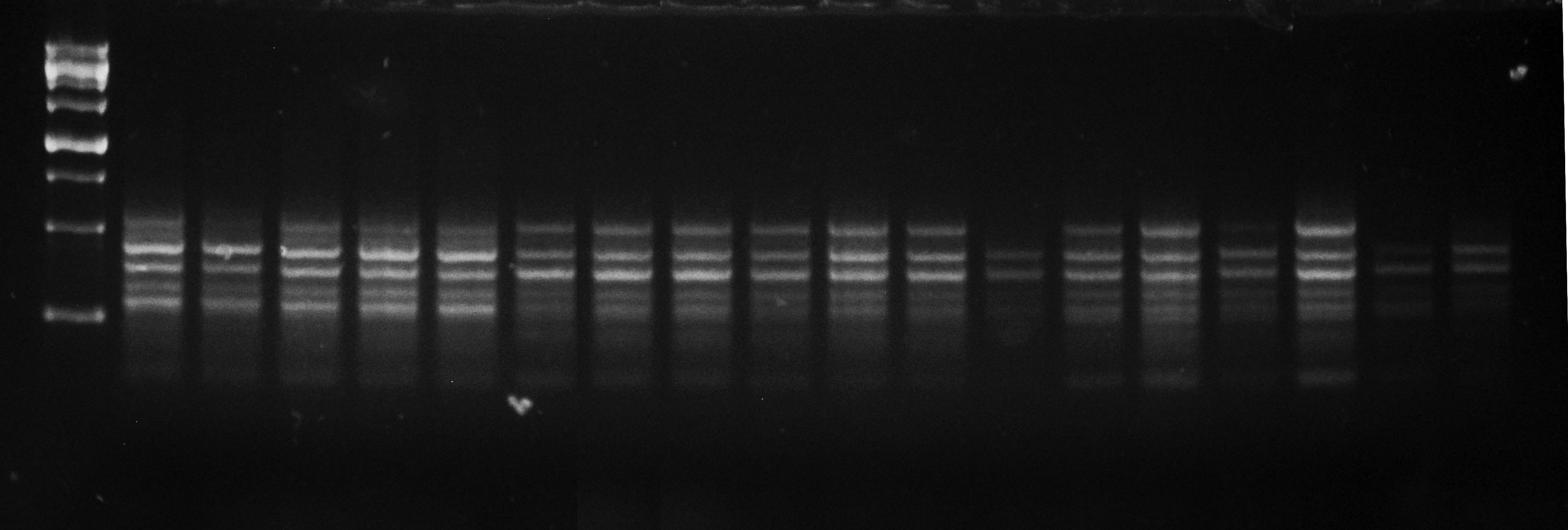


UBC 814


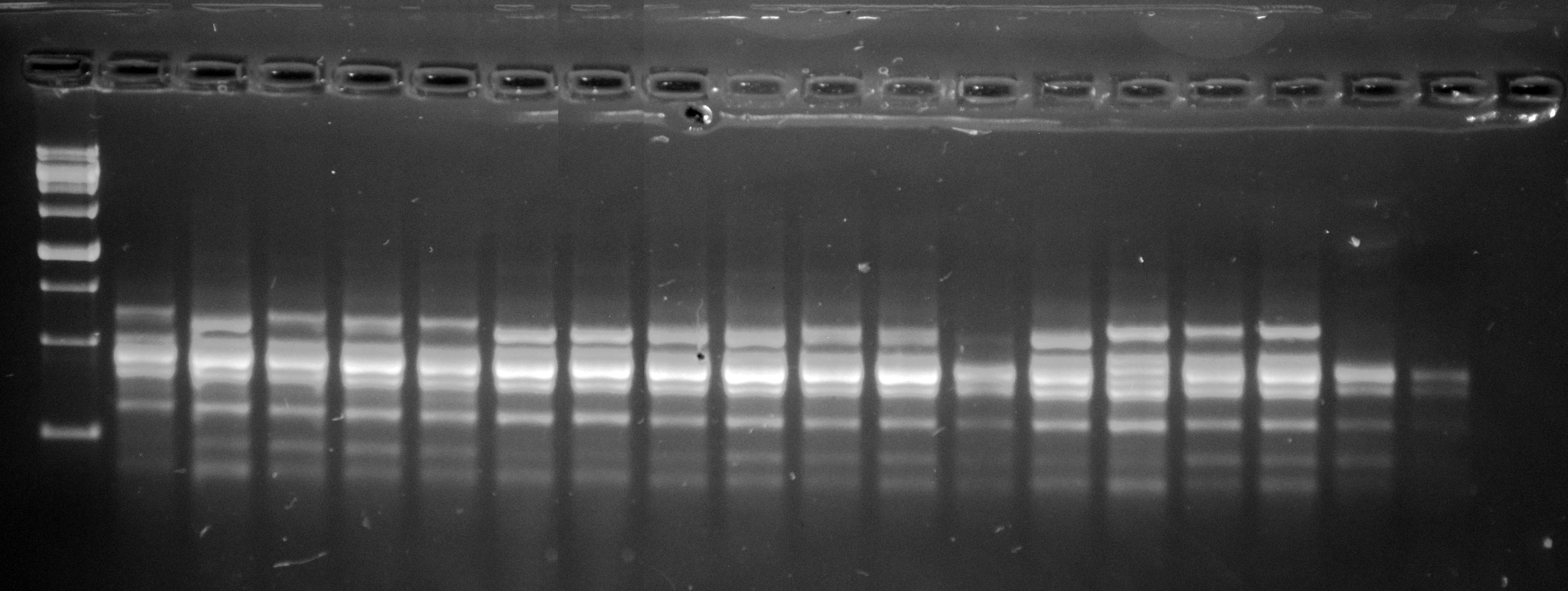


ISSR 835


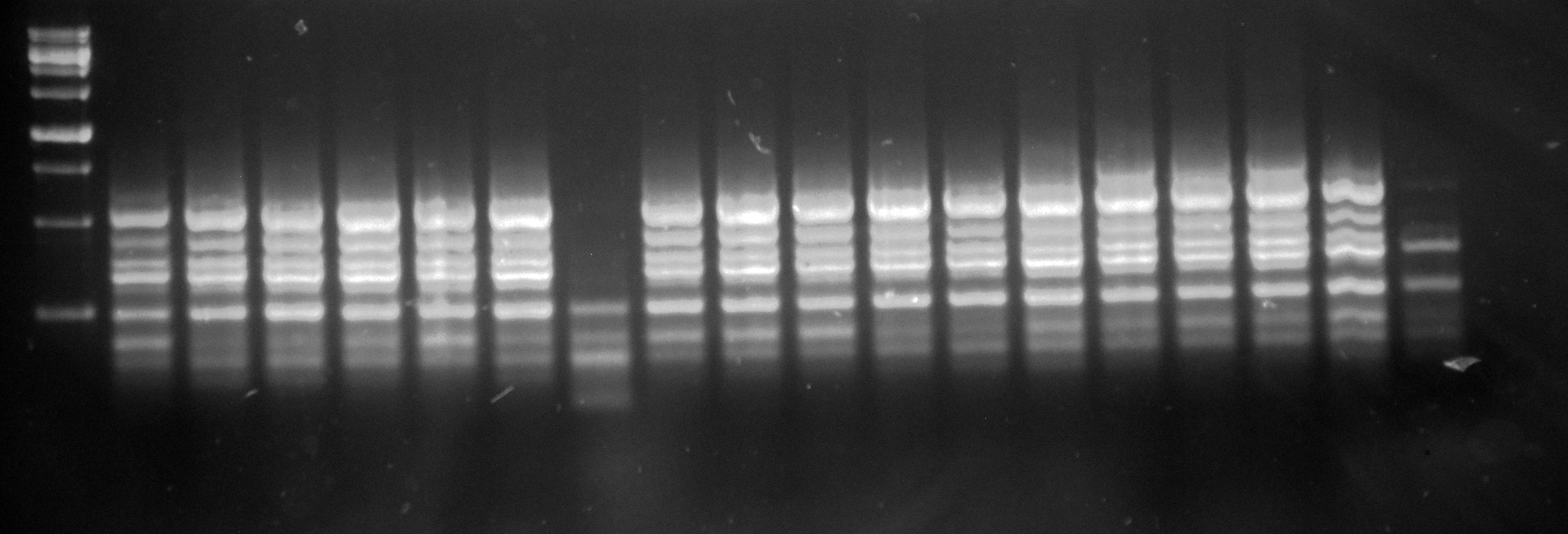


ISSR 825

**
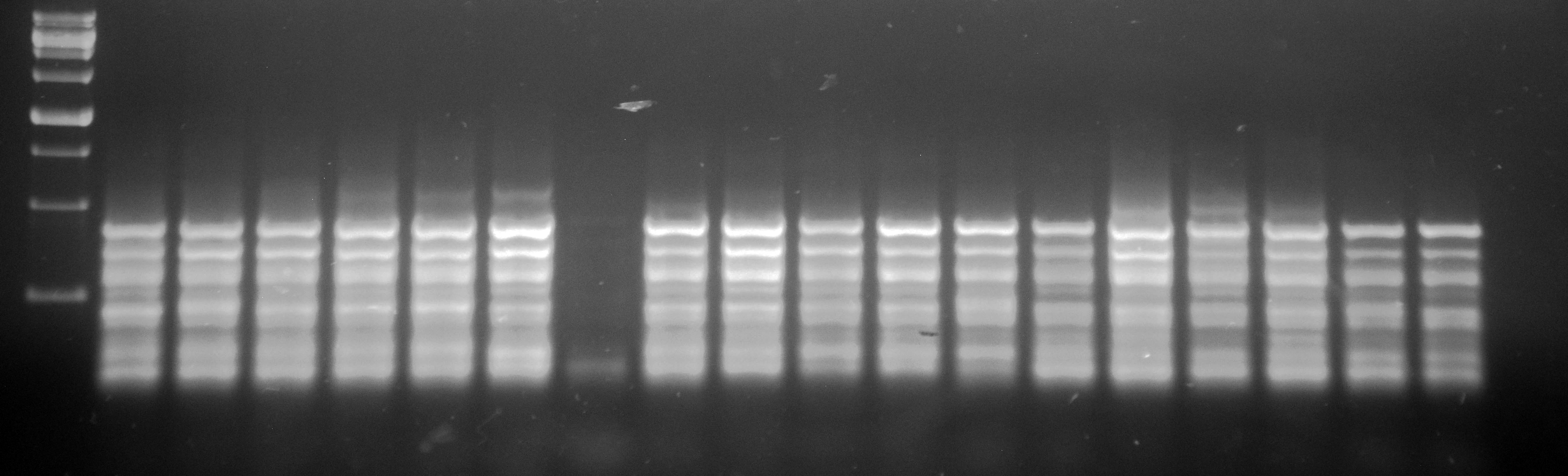
**

ISSR 901


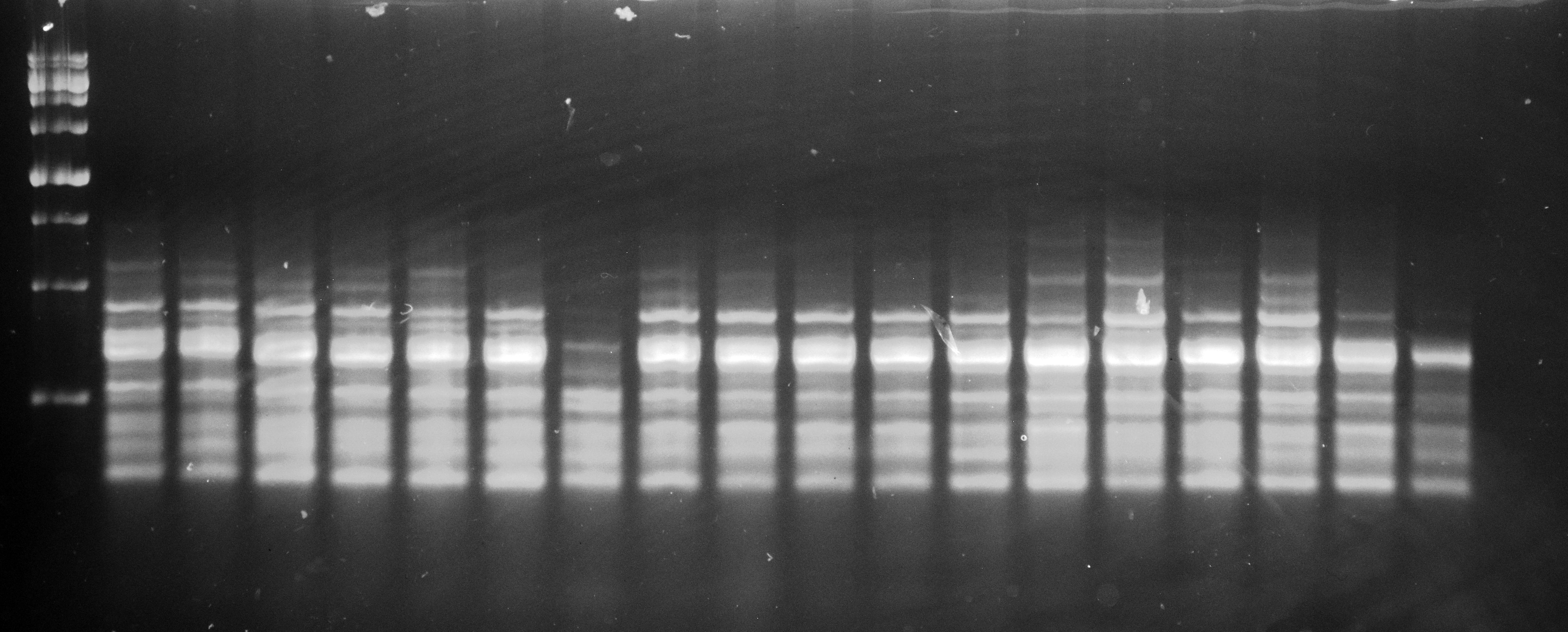


ISSR 810


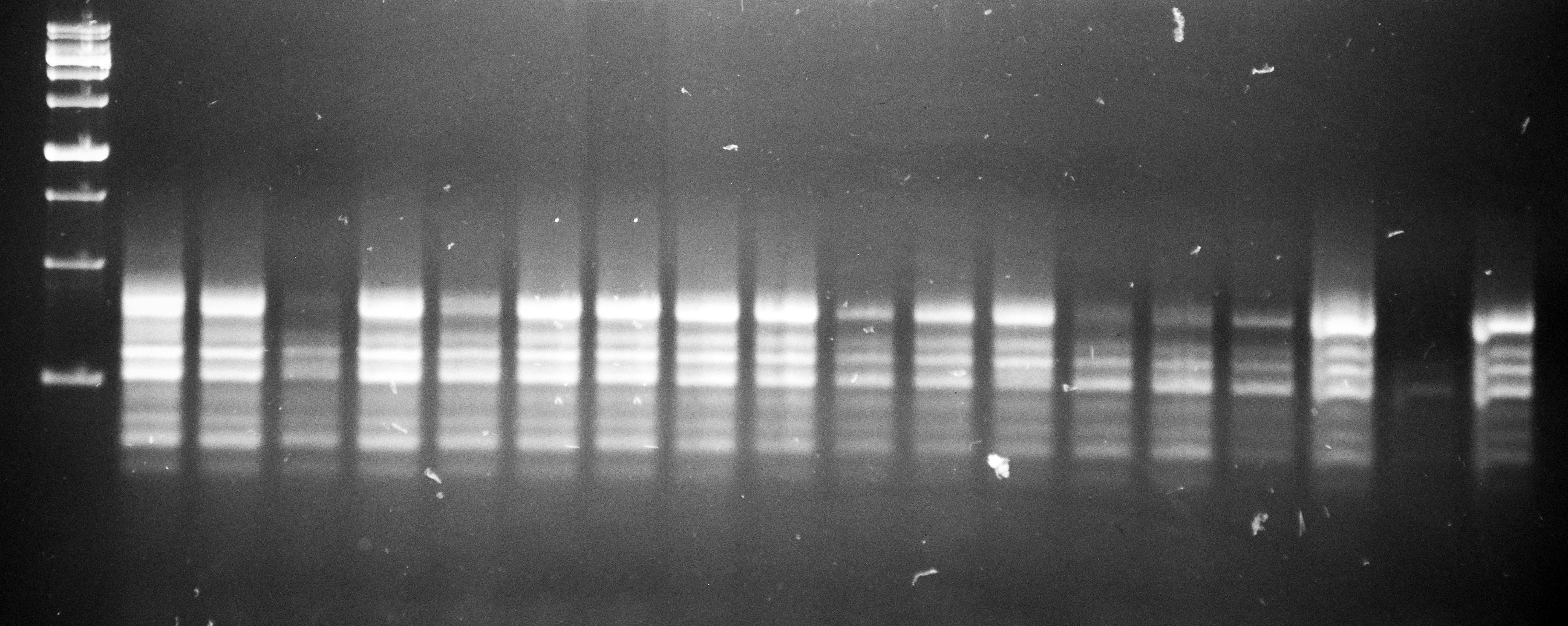


UBC 686


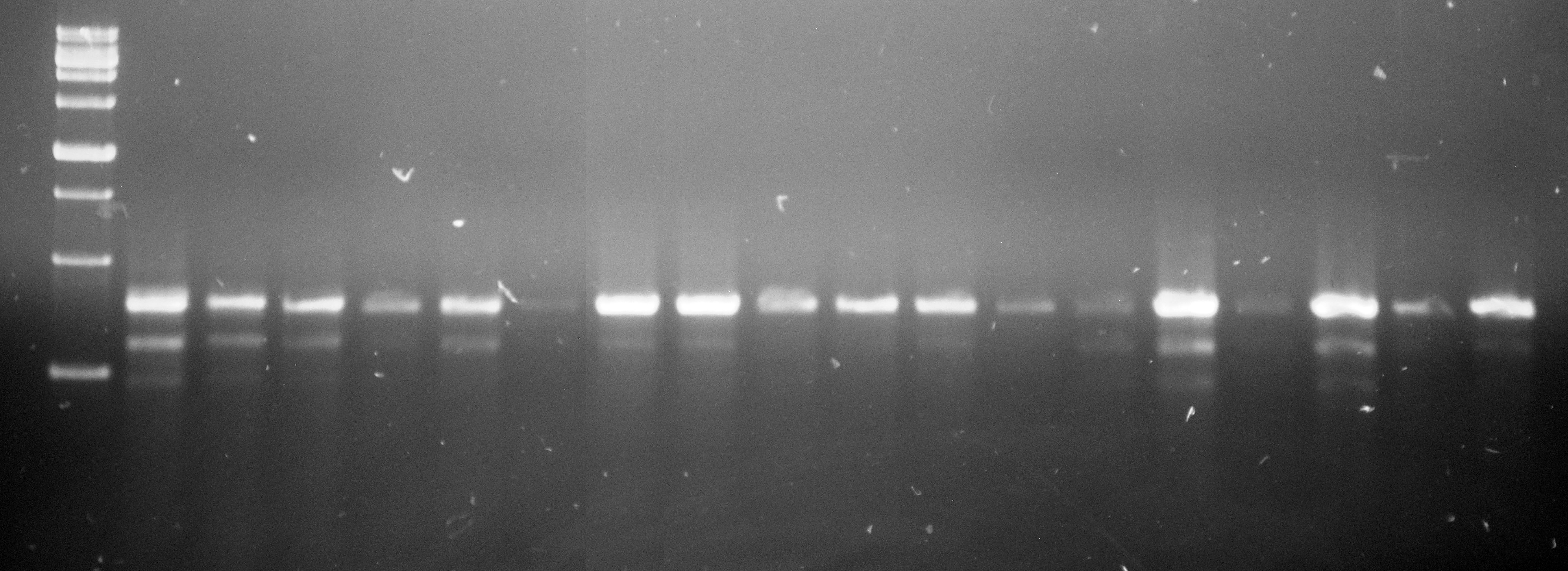


ISSR 807


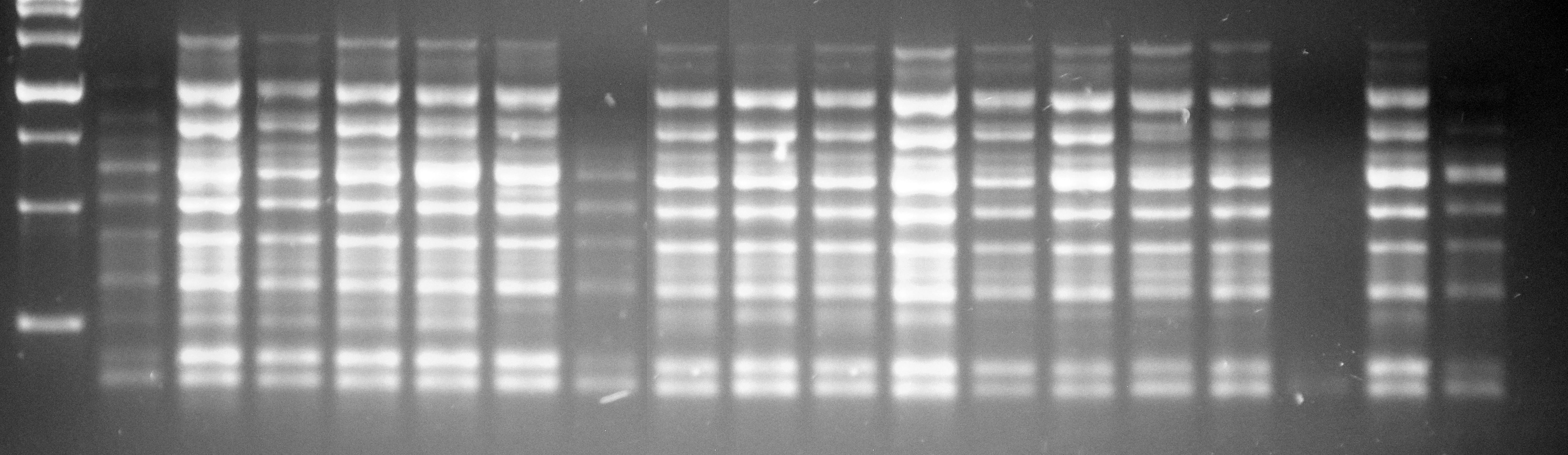


ISSR 857


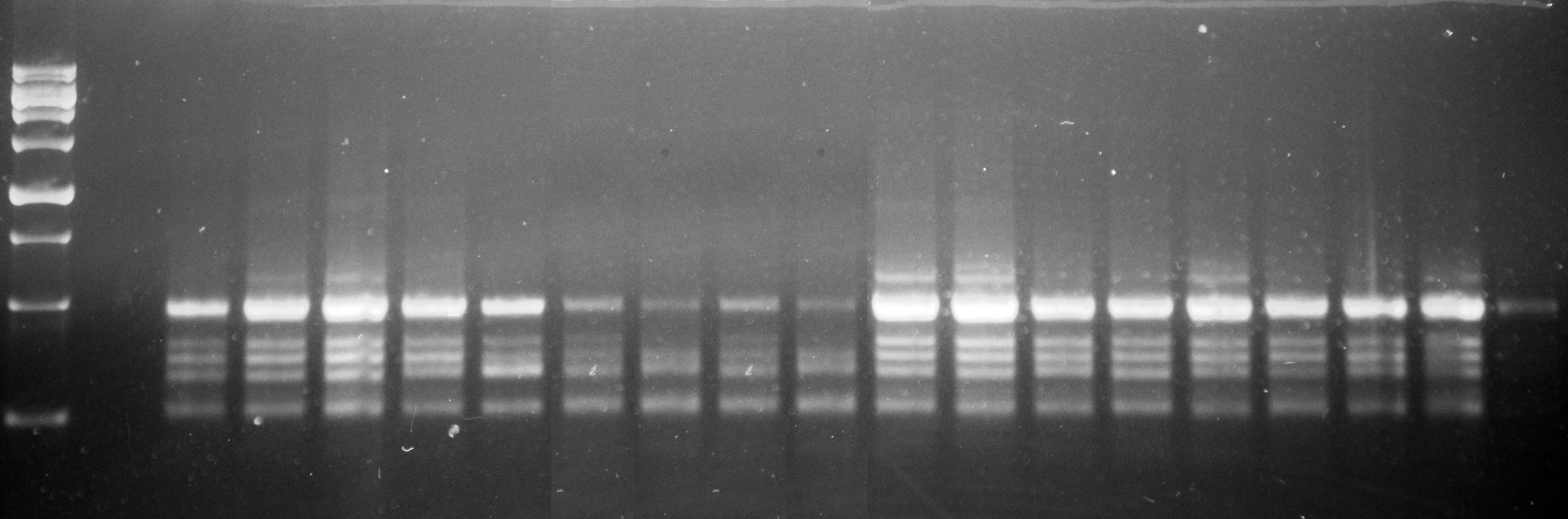


UBC 826


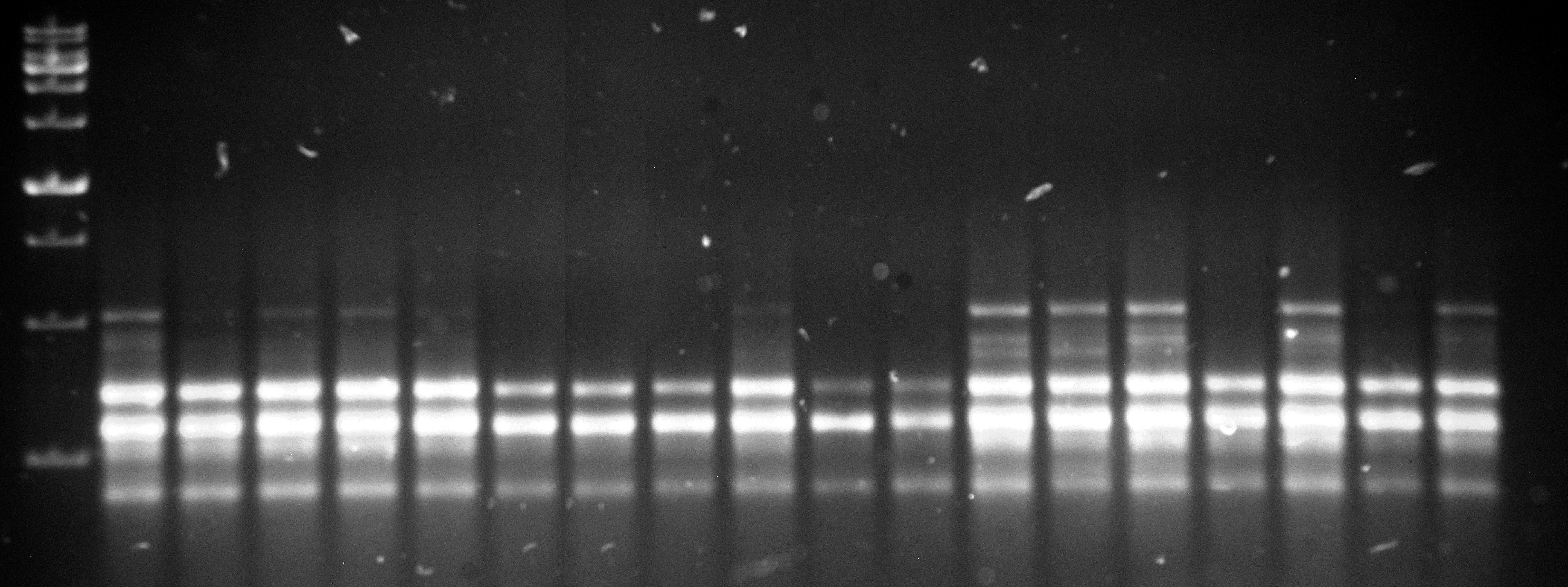


UBC 827


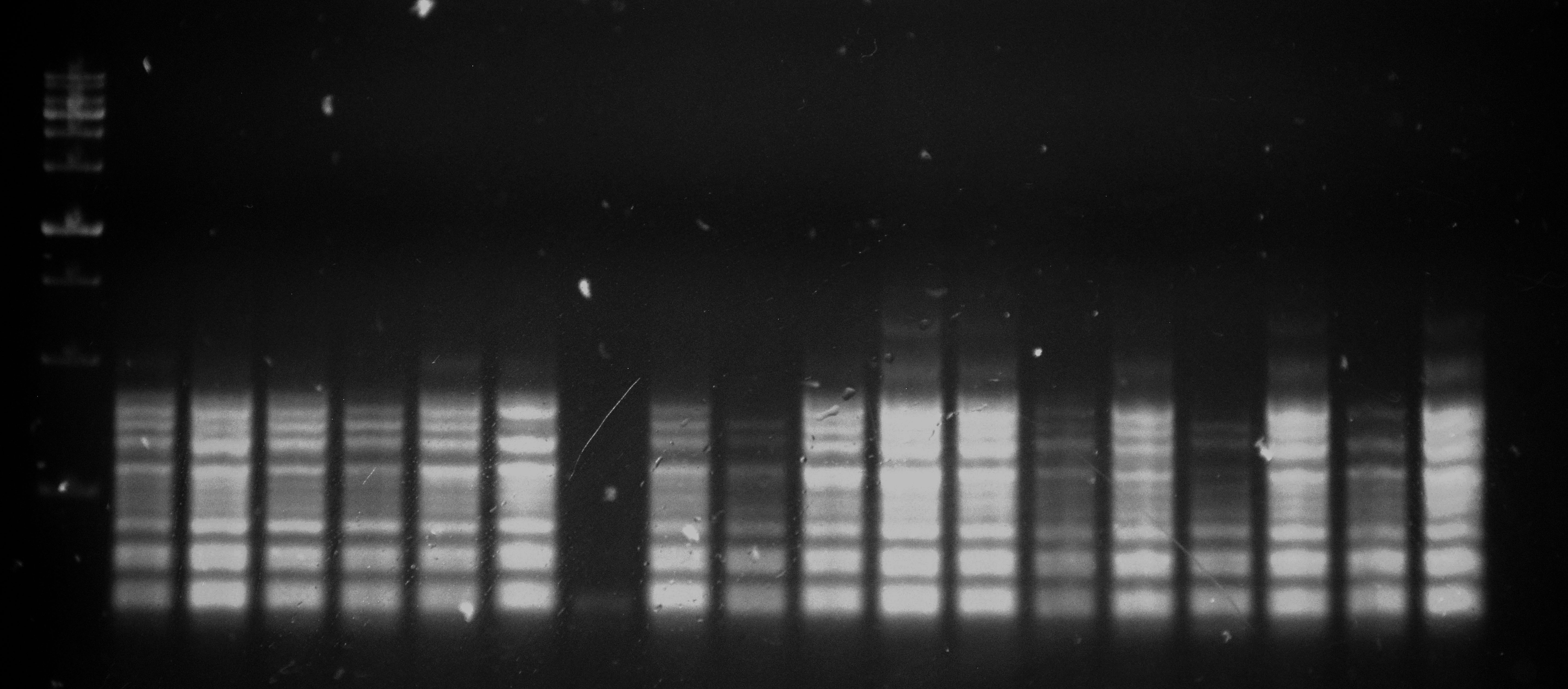


UBC 811
